# A Novel Ibuprofen Derivative and Its Complexes: Physicochemical Characterization, DFT Modeling, Docking, In Vitro Anti-Inflammatory Studies, and DNA Interaction

**DOI:** 10.3390/molecules27217540

**Published:** 2022-11-03

**Authors:** Abbas M. Abbas, Ahmed Aboelmagd, Safaa M. Kishk, Hossam H. Nasrallah, Warren Christopher Boyd, Haitham Kalil, Adel S. Orabi

**Affiliations:** 1 Chemistry Department, Faculty of Science, Suez Canal University, Ismailia 41522, Egypt; 2 Medicinal Chemistry Department, Faculty of Pharmacy, Suez Canal University, Ismailia 41522, Egypt; 3 Chemistry Department, Faculty of Dentistry, Sinai University, Kantara 41612, Egypt; 4 Chemistry Department, Cleveland State University, Cleveland, OH 44115, USA

**Keywords:** ibuprofen, anti-inflammatory, pharmacokinetic, DNA interaction, DFT, docking

## Abstract

A novel derivative of ibuprofen and salicylaldehyde N′-(4-hydroxybenzylidene)-2-(4-isobutylphenyl) propane hydrazide (HL) was synthesized, followed by its complexation with Cu, Ni, Co, Gd, and Sm. The compounds obtained were characterized by ^1^HNMR, mass spectrometry, UV-Vis spectroscopy, FT-IR spectroscopy, thermal analysis (DTA and TGA), conductivity measurements, and magnetic susceptibility measurements. The results indicate that the complexes formed were [Cu(L)(H_2_O)]Cl·2H_2_O, [Ni(L)_2_], [Co(L)_2_]·H_2_O, [Gd(L)_2_(H_2_O)_2_](NO_3_)·2H_2_O and [Sm(L)_2_(H_2_O)_2_](NO_3_)·2H_2_O. The surface characteristics of the produced compounds were evaluated by DFT calculations using the MOE environment. The docking was performed against the COX2 targeting protein (PDB code: 5IKT Homo sapiens). The binding energies were −7.52, −9.41, −9.51, −8.09, −10.04, and −8.05 kcal/mol for HL and the Co, Ni, Cu, Sm, and Gd complexes, respectively, which suggests the enhancement of anti-inflammatory behaviors compared with the binding energy of ibuprofen (−5.38 kcal/mol). The anti-inflammatory properties of the new compounds were assessed in vitro using the western blot analysis method and the enzyme-linked immunosorbent assay (ELISA), consistent with the outcomes obtained from docking. The half-maximal inhibitory concentration (IC_50_) values are 4.9, 1.7, 3.7, 5.6, 2.9, and 2.3 µM for HL and the Co, Ni, Cu, Sm, and Gd complexes, respectively, showing that they are more effective inhibitors of COX2 than ibuprofen (IC_50_ = 31.4 µM). The brain or intestinal estimated permeation method (BOILED-Egg) showed that HL and its Co complex have high gastrointestinal absorption, while only the free ligand has high brain penetration. The binding constants of Co, Cu, and Gd complexes with DNA were recorded as 2.20 × 10^4^, 2.27 × 10^6,^ and 4.46 × 10^3^ M^−1^, respectively, indicating the intercalator behavior of interaction. The newly synthesized ibuprofen derivative and its metal complexes showed greater anti-inflammatory activity than ibuprofen.

## 1. Introduction

Ibuprofen, an example of a non-steroidal anti-inflammatory drug (NSAID), shows therapeutic effects by inhibiting the cyclooxygenase (COX) enzyme (it inhibits both COX1 and COX2 isoforms) [1]. This enzyme catalyzes the manufacture of prostaglandins, which are endogenous mediators involved in the development of pain, inflammation, and fever [1,2]. A variety of derivatives of ibuprofen have been prepared, with modifications made to improve their analgesic and anti-inflammatory effects and minimize gastric side effects [3,4,5]. DNA binding tests of some ibuprofen derivatives revealed a strong relation with cancer cell (Huh-7) line activity, proposing that these compounds could be helpful to anti-cancer drug candidates [6]. Ibuprofen’s binary and ternary complexes showed antibacterial activity and enhanced anti-inflammatory behavior, as in its complexes with ruthenium, vanadium, and silver [7,8,9].

Schiff bases have attracted considerable attention from organic and medicinal researchers because they exhibit diverse biological and pharmaceutical applications. Hydrazones have pronounced biological and pharmaceutical activity in medicinal chemistry, such as antimicrobial [10], antibacterial [11], antifungal [12], antiviral, antimalarial [13], antitumor [14], anticancer [15,16], anti-tubercular [17,18], anti-inflammatory [19], anticonvulsant [20] and antiplatelet [21] activities. They are also used as catalysts in the polymer and dyes industry, besides some uses as antifertility and enzymatic agents [22].

A number of Schiff bases and related compounds derived from ibuprofen (imides, hydrazones, and hydrazides) have been synthesized, and showed unusual chemotherapeutic behavior [23,24]. The metal complexes formed from certain Schiff bases have many applications in the pharmaceutical, medicinal, agricultural, and industrial fields, with activity and effectiveness often exceeding that of its individual Schiff bases or free metal ions [25,26,27].

Based on previous work showing the medicinal potential of ibuprofen derivatives, the main goal of our work is to synthesize a further series of ibuprofen derivatives, based on the hypothesis that some modifications may result in a compound or compounds with better anti-inflammatory activity and a more favorable side-effect profile. The modification strategy was as follows: ibuprofen hydrazide was condensed with salicylaldehyde, resulting in a new ibuprofen compound which was then complexed with copper(II), nickel(II), cobalt(II), gadolinium(III), and samarium(III). Various techniques were used to characterize the obtained ligands and their complexes. The physicochemical characteristics were tested both in silico and experimentally. The docking was carried out by targeting the protein of COX2 (PDB code: 5IKT, Homo sapiens) to estimate, theoretically, the biological behavior of the compounds. The pharmacokinetic properties of HL and the cobalt complex were incorporated into the online SwissADME web tool. The in vitro evaluation of ibuprofen derivatives and their complexes using the enzyme-linked immunosorbent assay (ELISA) and western blot analysis against COX2. Based on these preliminary theoretical and experimental results, these novel species have potential as drug candidates, and future work will involve in vivo studies using a mouse model.

## 2. Results and Discussion

### 2.1. Description of HL

TLC on silica gel plates with the appropriate solvent system (90% chloroform:10% ethanol) was used to check the purity of the new compounds and the reaction completion.

Ibuprofen ester (EI) and its hydrazide (HI) were prepared as mentioned in the parts of the experiment. The ligand HL (C_20_H_24_N_2_O_2_) was designed by the condensation of ibuprofen hydrazide with salicylaldehyde, giving an excellent yield (85%).

HL is a canary-yellow crystalline product with a melting point of 159 °C. The ligand solubility was examined in various solvents, and it was shown to be soluble in DMSO, DMF, ethanol, methanol, and acetonitrile. The elemental analysis of the HL confirmed the empirical chemical formula with the values C% = 74.05 (74.10); H% = 7.46 (7.51); N% = 8.63 (8.55), calc. (found), respectively (Table 1).

**Figure 1:**
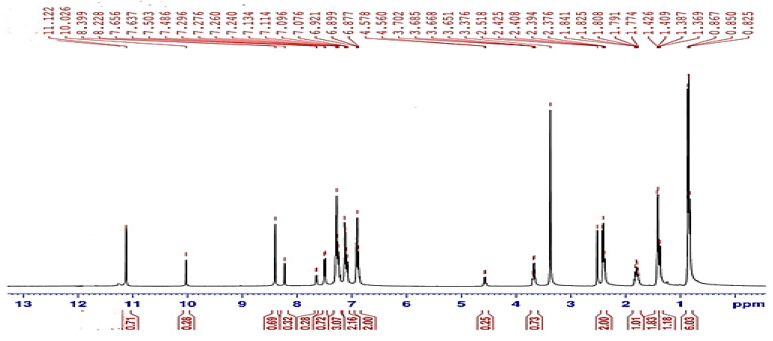
^1^HNMR spectrum of HL ligand.

The ^1^HNMR spectra were acquired for the synthesized compounds in the DMSO-d_6_ solvent. The ^1^HNMR spectral data of HI and the HL Schiff base are illustrated in Figure 1, Table S1 and Figure S1. 

The ^1^HNMR spectra revealed a singlet peak related to the (−NH_2_) group of the HI that appeared at 4.18 ppm. The ^1^H NMR for HL (in DMSO-d6) exhibited the appearance of some protons as two sets of signals for each proton of -CONH-N, -N=CH, Ar-CH-CO and some Ar-H groups as shown on the integration of the signals (Figure 1). The -CONH-N proton appeared as pair of singlet signals: the first at 11.12 ppm and the second at 10.02 ppm. In addition, the azomethine proton (N=C−H) has been shown as two singlet peaks at 8.39 ppm and at 8.22 ppm. Analogously the aromatic proton signals appeared as two doublets at 7.65 and at 7.50 ppm, and the proton of Ar-CH-CO group was shown as two multiples at 4.57 and 3.70 ppm. These two sets of ^1^HNMR signals of HL were explained by the existence of this hydrazone as an equilibrium mixture from cis-E and trans-E conformers in DMSO solution as described in previous literature of similar hydrazones [28,29,30].

**Figure 2:**
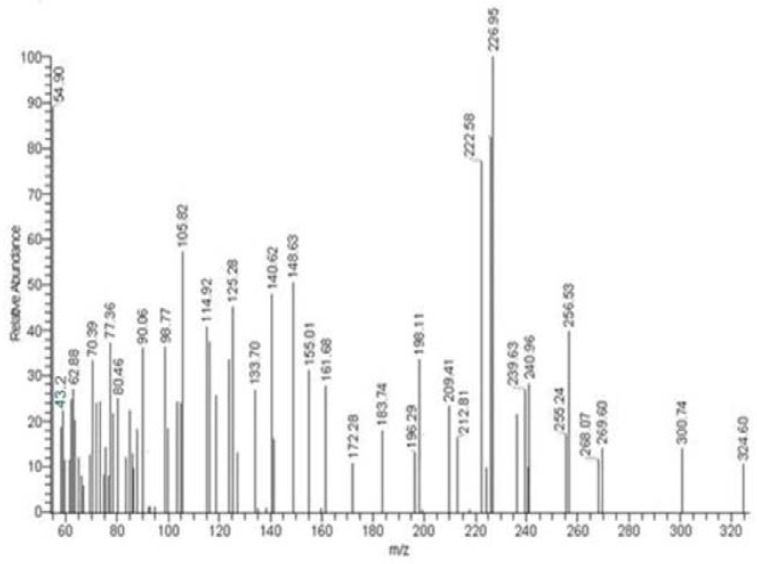
The HL ligand’s mass spectra.

The one-peak chromatogram indicated the purity of the HI and HL ligands (Figure 2 and Figure S2). The mass spectrum confirms the ligand-reported empirical formula. The molecular ion peak appears at *m*/*z* = 221.11 (11.02%) and 324.60 (10.28%) for HI and HL, respectively, identical to the molecular weights of the target ligands. The stepwise ligand fragmentation was proposed and approved in (Schemes S1 and S2). The obtained fragments are consistent with the reported results, indicating that the target compound’s molecular formula is correct.

**Figure 3:**
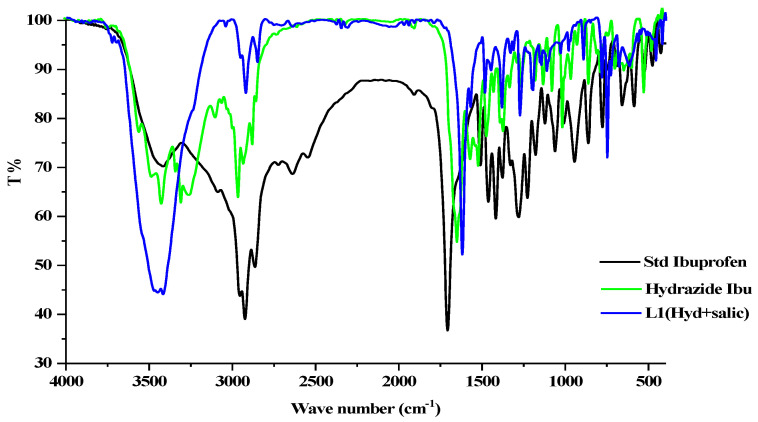
FTIR spectrum of the ligand HL, HI, and ibuprofen.

The FT-IR spectrum of pure ibuprofen appears with a robust, broad band at 3416 cm^−l^ because of hydroxyl stretching vibration; the band’s broadness may arise from inter-hydrogen bonding. C=O stretching is related to the peak at 1709 cm^−l^ (Figure 3). Ibuprofen hydrazide shows a fork-shaped band with maxima at 3300 and 3265 cm^−1^ assigned for −NH_2_ vibrations; meanwhile, (−NH) stretching appeared around 3420 cm^−1^. The ketone group (C=O) appeared at 1638 cm^−1^; the decreases in frequency for the ketone band may result from the tautomerism structure, which produces a weak band at 3555 cm^−1^ for υ(OH) (Figure 3) [31].

The HL ligand’s significant infrared frequencies are displayed in Figure 3 and Table 2. The stretching vibration of υ(OH) occurs as a broad band centered at 3451 cm^−1^. The existence of inter- or intramolecular hydrogen bonds could explain the broadness of these bands [32]. A robust and sharp band at 3418 cm^−1^ is related to the stretching vibration of the NH group [33]. The band at 1622 cm^−1^ could be related to υ(C=O) [34]; meanwhile, the azomethine group υ(C=N) appeared at 1576 cm^−1^ [35]. The absence of υ(NH_2_) vibration bands and observation of the υ(C=N) band in FTIR spectra helped confirm the formation of ligand HL.

The UV-vis spectrum of the HL appears in three bands at 280, 290, and 322 nm, as shown in Figure S3. These bands showed a blue shift in methanol solvent, indicating the π → π* and n → π* electronic transitions.

The TG and DTA analysis of the HL was done to examine the ligand’s thermal decomposition behavior. The thermal assessment was done in a nitrogen atmosphere with a 10 °C/minute heating; over a temperature range of 200 to 600 °C. The thermal decomposition of the Schiff base reveals that mass loss occurs in several steps. The TGA and DTA data for HL are shown in Figure S4.

The thermogram of HL shows two decomposition steps in the ranges of 294–341 °C and 476–520 °C, with 317 and 478 °C midpoints and mass loss of 86.4% and 12.6%, respectively, without any residue (650 °C). All thermal breakdown steps have exothermic behavior with ΔH = 0.363 and 1.41 kJ/g, respectively [36]. The DTA data showed an endothermic peak at 159 °C (ΔH = 36.1 J/g) accompanied by zero weight loss, confirming the melting point of the synthesized ligand.

The organic molecule HL has thus been thoroughly characterized by spectroscopic and thermal techniques, which provide evidence of its purity and suitability as a synthon for coordination complexes of d-block and f-block metals.

### 2.2. Characterization of Metal Complexes

The Cu(II), Ni(II), Co(II), Gd(III), and Sm(III) ions interact with the HL ligand to form the complexes [Cu(L)(H_2_O)]Cl·2H_2_O (dirty green), [Ni(L)_2_] (apple green), [Co(L)_2_]·1/2H_2_O (brick brown), [Gd(L)_2_(H_2_O)_2_](NO_3_)·3/2H_2_O (yellow-orange) and [Sm(L)_2_(H_2_O)_2_](NO_3_)·2H_2_O (buff). The formed complexes are crystalline and soluble in DMSO solvent, and they did not melt up to 280 °C.

The electric conductivity of produced compounds was assessed in DMSO (0.001 M), which implies the electrolytic character of the synthesized compound with behavior resembling the 1:1 electrolyte, except for the non-electrolytic Co and Ni complexes. Physical features of the ligand HL and its metal complexes, conductivity, and CHN and M percent data were reported in Table 1. Two analytical methods, complexometric titration and thermogravimetry, were used to determine the M% concentration. The results supported the previously assigned formulas [37,38].

#### 2.2.1. FTIR Spectra

**Figure 4:**
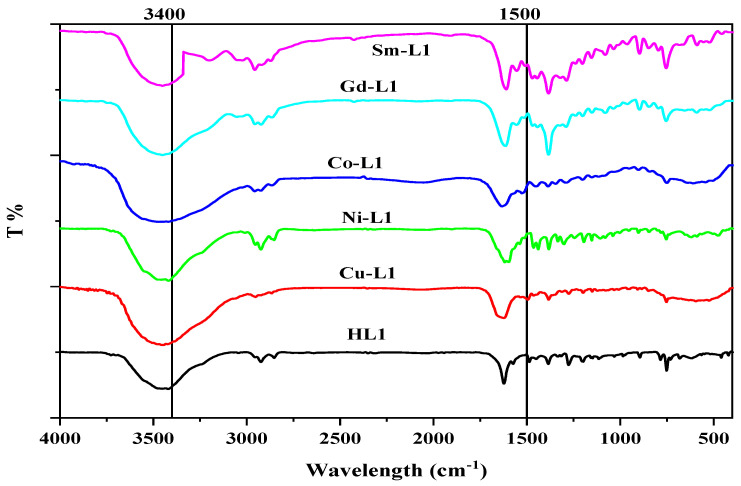
FTIR spectrum of HL ligand and its metal complexes.

The IR spectra of the complexes were evaluated against those of the ligand HL to detect the coordination sites implicated in complexation. Chelation is likely to modify peak position and/or intensities. The synthesized compounds’ infrared spectra are shown in Table 2, Figure 4 and Figures S5–S9.

The firm broadband at 3500–2900 cm^−1^ is described as the stretching vibration of υ(OH) and υ(NH). The band around the 3800–3660 cm^−1^ range may be consequential to the existence of water (coordinated and/or outer-sphere crystalline) in the synthesized complexes [39,40]. The robust broadband of [Ni(L)_2_] over the 3800–3030 cm^−1^ due to the resonated form, which brings O-H, which might be produced inter- and intra- hydrogen bonds [39]. The C=O appeared at 1622, 1627, 1603, 1632, 1612, and 1609 cm^−1^ for HL and Cu(II), Ni(II), Co(II), Gd(III), and Sm(III) complexes, respectively. On complexation, this band shifted, confirming the participation of this group in chelation [41,42,43]. The changes in the band appeared at 1576, 1539, 1520, 1530, 1528, and 1539 cm^−1^ for HL and Cu(II), Ni(II), Co(II), Gd(III), and Sm(III) complexes, respectively, which ascribed to the azomethine group υ(C=N) stretching vibration, confirm the association in complexation.

A sharp and robust band, which presents around 1384 cm^−1^ and a weak band around 1441 cm^−1^ in the case of Gd(III) and Sm(III) complexes, could be ascribed to the vibration of the nitrate group. The different between υ_1_ and υ_4_ of nitrate is 60 cm^−1^, reflecting nitrate groups’ ionization or monodentate behavior [44,45].

The metals complexes produced weak bands at 653, 612, 608, 641, and 588 cm^−1^ assigned to the M-O stretching band; meanwhile, the M-N band appeared at 580, 584, 545, 584, and 578 cm^−1^ for Cu(II), Ni(II), Co(II), Gd(III) and Sm(III) complexes, respectively.

**Table 2:** Significant IR frequencies (cm^−1^) for HL Schiff base and its complexes.

Compound	υ(H_2_O, OH, NH)	υ(C=O)	υ(C=N)	υ NO_3_	υ M-O	υ M-N
HL	3500–2900 m, br	1622 w	1576 s	---	---	---
[Cu(L)(H_2_O)]Cl.H_2_O	3740–3000 m, br	1627 w	1539 sh	---	653 w	580 w
[Ni(L)_2_]	3800–3030 m, br	1603 w	1520 sh	---	612 w	584 w
[Co(L)_2_]1/2H_2_O	3830–2990 m, br	1632 w	1530 sh	---	608 w	545 w
[Gd(L)_2_(H_2_O)_2_](NO_3_).3/2H_2_O	3800–2990 m, br	1612 w	1528 sh	1384 s	641 w	584 w
[Sm(L)_2_(H_2_O)_2_](NO_3_).2H_2_O	3700–3100 m, br	1609 w	1539 sh	1384 s	588 w	578 w

s: strong, m: medium, w: weak, br: broad, and sh: shoulder.

#### 2.2.2. Thermal Analysis

**Figure 5:**
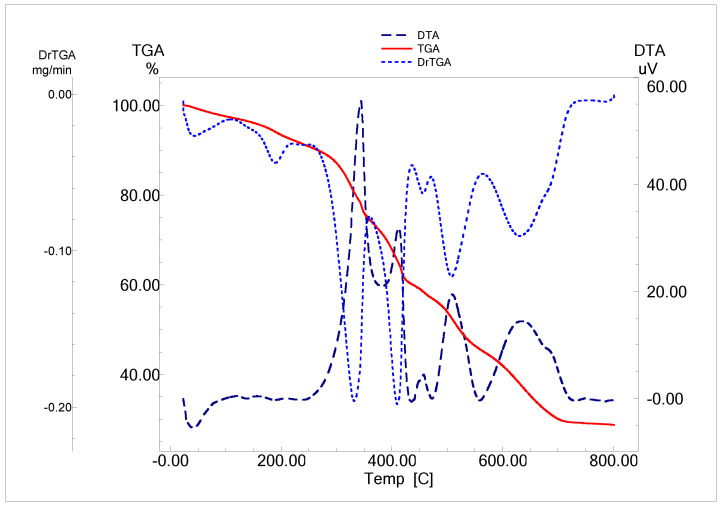
TGA/TG and DTA curves of [Gd(L)_2_(H_2_O)_2_](NO_3_).3H_2_O complex.

The estimated weight loss, decomposition steps, temperature ranges, and decomposition products of the complexes are presented in Figure 5 (as example), Table 3 and Figures S10–S13.

[Co(L)_2_]·1/2H_2_O, [Cu(L)(H_2_O)]Cl·H_2_O, [Gd(L)_2_(H_2_O)_2_]NO_3_·3/2H_2_O and [Sm(L)_2_(H_2_O)_2_(NO_3_)]NO_3_·2H_2_O, respectively, dehydrated at 64, 69, 73, and 83 °C. This step showed mass loss percent of 1.25, 3.68, 2.83, and 3.65% (Calc. 1.31, 3.93, 2.91, and 3.87%), accompanied by endothermic DTA peaks at 60, 55, 116, and 128 °C, respectively. The initial step involves the loss of water of crystallization, except for [Ni(L)_2_].

The second decomposition step (the first, for the Ni complex), indicates the decomposition of the target Schiff base and the evolution of other coordinated molecules (water, HCl, or HNO_3_) (Table 3). Broad DTG bands appear with maxima at 337, 234, 250, 190, and 329 °C for Co, Ni, Cu, Gd, and Sm, respectively. The observed mass decrease was 40.58 (40.71), 15.35 (15.67), 46.62 (46.45), 3.9 (3.76), and 27.49 (27.56) (Found/Calc. %).

The final decomposition step for Co and Cu complex has a DTG peak at 473 and 606 °C with mass loss of 46.63 (46.39) and 32.8 (32.66)%.

The third decomposition step (the second, for the Ni complex), indicates the continued ligand decomposition and/or liberation of HNO_3_. This step has a DTG peak at 337; 371, 330; 405, and 441 °C for Ni, Gd, and Sm. The observed mass reduction was 46.25 (46.21), 32.1 (32.5), and 23.37 (23.38) (Found/Calc. %). The nitrate group was liberated in Gd and Sm complexes at this step.

The final step of the decomposition occurred at 449, 510, 630, and 499 °C for Ni, Gd, and Sm complexes. At this step, the observed mass reduction was 28.4 (28.26), 29.7 (30.03), and 21.21 (21.58) (Found/Calc. %).

The residue agrees well with the determined value and the proposed formulas as CuO, NiO, and CoO; meanwhile, in the case of Gd and Sm complexes, the residue has some undecomposed ligands (carbonaceous residue).

All the steps after dehydration showed an exothermic behavior; all data, including peak temperature and enthalpy change, are in Table S2.

#### 2.2.3. Thermodynamic and Kinetic Parameters

The order (n), pre-exponential factor (Z), and heat of activation (E_a_) of the different degradation steps were estimated from the TG and DTG thermograms by using the Coats-Redfern equation to analyze the influence of ligand structural features on the complexes’ thermal behavior [46,47]. Examples of the complex linearization curves were produced using the Coats-Redfern equation in Figures S14–S18. The decomposition steps were chosen based on their appearance and intensity. Table 4 shows the investigation results of the kinetic parameters ΔG*, ΔH*, and ΔS* for the decomposition stage (Table 4).

The temperature of the reaction, among other factors, influences the frequency of collisions. When the temperature rises, the particles’ average rate rises as well. These particles’ average kinetic energy is also rising. The activation energy (E_a_) is needed for all chemical processes, including exothermic reactions. Reactant molecules need activation energy to approach one another, resist repulsion forces, and begin weakening bonds.

According to the collision theory, we can discuss our results as the following:

1. The slight variation in ΔS* revealed the near constancy of the disorder through the thermal decomposition reactions. The structure of the activated complexes is more ordered than that of the reactants, and the negative values show that the reactions are slower than usual.
2. The Cu complex produced the highest pre-exponential factor (Z) for the ligand decomposition (6.41 × 10^10^ s^−1^) in addition to the most significant Ea (184.05 J/mol);
3. The selected decomposition steps have negative ΔH*, indicating these processes’ exothermic behavior.
4. ΔG* has a positive value for all complexes under consideration, with three exceptions with negative values, cited in Table 4. The positive values revealed the non-spontaneous behavior according to the ΔG* concept.
5. The moderate Ea value hints at these compounds’ reactivity in thermal decomposition reactions.

#### 2.2.4. UV-Vis Spectra and Magnetic Moment

**Figure 6:**
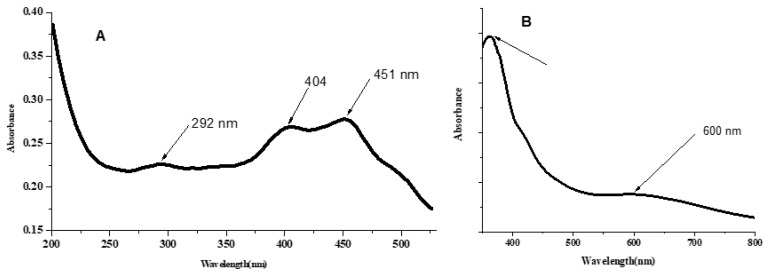
UV-Vis spectra (**A**) L-Cu complex in DMSO solution (1 × 10^−5^ M); (**B**) Nujol mull of L-Cu complex (solid complex suspended in paraffin oil).

In addition to their assignments, the spectral measurements of the target complexes (in DMSO solutions and nujol mull) are summarized in Table S3, seen in Figure 6 and Figures S17–S21. The electronic transitions attributed to the π→π* and n→π* for HL ligand at 280, 290, and 322 nm involve hypsochromic (blue shift), which reflects the ligation with the metal ion in the complexation [48]. The nujol mull method has been used to obtain the visible spectrum of the solid complex, to maximize the absorption for d-d transition bands [49].

The Cu complex has a magnetic moment of 1.49 BM, indicating a square planar or tetrahedral structure. The low magnetism value could be caused by a bond between two Cu ions and/or the formation of some orbital overlap of the d-p interaction type of metal ion and ligand. The absorption bands appeared at 363 and 292 nm due to n→π* and π→π* transitions [48]. The bands at 451 and 404 may be assigned to ligand to metal charge transfer. Broadband around 600 nm with a very low molar extinction coefficient may be assigned to ^2^B_1g_→^2^B_2g_ transitions, resulting from the d-d transition in a square planner environment [50].

The paramagnetic Ni complex exhibited octahedral geometry with a magnetic moment of 3.24 BM. The electronic spectrum graph shows 317, 397, 402, and 628 nm absorption bands related to n→π*, CT, and d→d transitions. The d-d transition gave the characteristic bands for octahedral structure and could be assigned the transition bands as the following: 3A2g(F) → 3T2g(F), 3A2g(F) → 3T1g(F), and 3A2g(F) → 3T1g(P).

On the other hand, the Co complex shows a magnetic moment value of 5.0 BM, indicating a high spin complex with hybridization sp3d2. The UV-Vis spectra of the cobalt complex exhibit a strong band at 290 nm related to the π → π* transition. The bands that appeared at 407 and 450 may be assigned to CT. The d-d transition bands observed at 506 nm are characteristic of the d-d transition 4T1g(F) → 4T2g(P), which belongs to the spin-allowed but LaPorte-forbidden transition [51].

The magnetic moments for Gd(III) and Sm(III) complexes are 8.97 and 1.33 BM, which are very similar to those seen for high-spin bicapped trigonal prismatic/square antiprismatic structures. The Gd(III) compounds’ UV-Vis spectra revealed absorption bands at 291, 402, 452, and 504 nm as π → π*, n→π*, and CT transition; meanwhile, Sm(III) complex shows bands at 239 and 347 nm as π→π*and n→π* transition [52,53].

The difference between bands observed for the produced complexes and the free ligand supports the formation of the complexes under discussion. The metal bands reflect on being obscured by charge transfer bands or ligand bands of high intensity. The Dq values for Co, Ni, and Cu complexes presented in Table S3 are significantly varied [54,55].

**Scheme 1:**
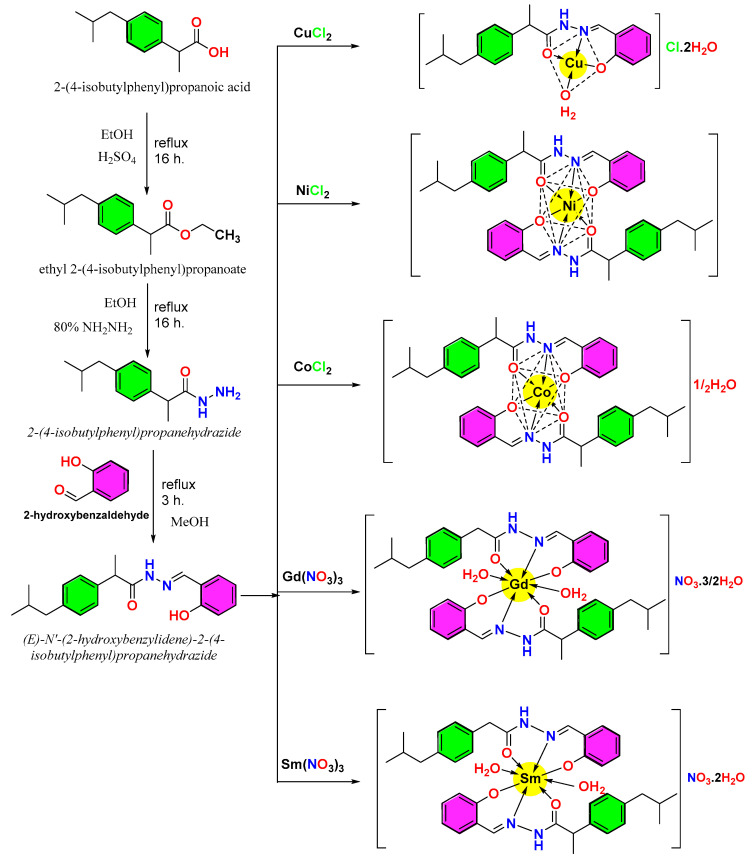
The preparation of the HL and its complexes postulated structures.

Based on elemental analysis, FTIR, thermal analysis, magnetic features, UV-visible spectra, and molar conductivity, the predicted structures of the complexes are illustrated in (Scheme 1). 

**Scheme 2:**
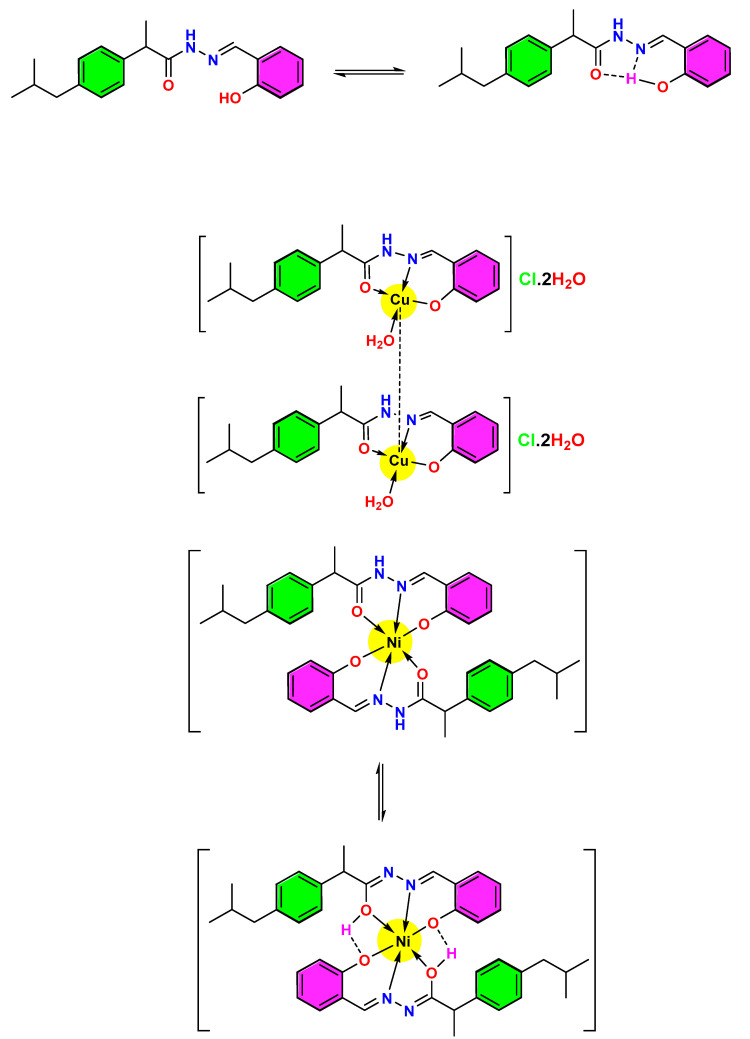
The suggested resonated form of HL ligand, Ni, and Cu complexes (coupling between two Cu ions).

The tautomeric structure of the HL ligand and Ni complex, which can explain the presence of the firm and broad band of O-H in the FTIR spectra and the Cu complex with the Cu-Cu delta bond, is shown in Scheme 2.

The spectroscopic, thermal, and magnetic measurements of the complexes discussed above provide strong evidence for their proposed structures and allow for the use of these compounds in in-vitro biological studies, as described in Section 2.6 and Section 2.7 below. In addition, the proposed structures were used in computational studies, as described in Section 2.3, Section 2.4 and Section 2.5.

### 2.3. Computational Chemistry and Molecular Modeling

Computational modeling of drug candidates can be used to predict their biological availability based on electron density and hydrophilicity vs. lipophilicity. Biological activity can be predicted and observed as a biological activity rationalized by docking studies, modeling interactions between drug candidate molecules and target biomolecules such as proteins. The electron density, surface characteristics, and frontier molecular orbitals of ibuprofen, the novel compound HL, and its metal complexes were modeled using density functional theory (this section), and their interactions with the COX2 protein were modeled with docking studies (Section 2.4). In addition, the BOILED-Egg model was used to predict the ability of ibuprofen, HL, and the cobalt complex L-Co to be absorbed through the gastrointestinal system and pass the blood-brain barrier (Section 2.5).

#### 2.3.1. The Surface Characteristics of the Formed Compounds

The surface features of the compounds are the most valuable features that influence drug manufacturing. The drug’s action with living cells shows the presence of an active lone pair and its lipophilic nature. The lipophilic maps of the formed compounds are shown in Table 5. The active lone pair map was a color scheme: violet = H-bonding, green = Hydrophobic, and blue = Mild Polar. For HI and HL, H-bonding ability was localized in three locations. The hydrophobic characteristics complement the capacity to form H-bonds. The lipophilic map has several colors, including violet (hydrophilic), white (neutral), and green (lipophilic) [56,57].

#### 2.3.2. The Ligand Properties and Density Function Theory (DFT)

A study of the highest occupied molecular orbital (HOMO) and lowest unoccupied molecular orbital (LUMO) energy and DFT calculations were carried out for Ibuprofen, HI, and HL. Each compound was submitted to energy shift using the force field parameter MMFF94x. The simulation procedure was done using the SCF (self-consistent field method, commonly known as the Hartree–Fock method) computation. MOPAC was utilized with Hamiltonian PM3 and the RHF (limited Hartree-Fock approach).

##### Calculation of the Parent Drug’s Density Function

**Figure 7:**
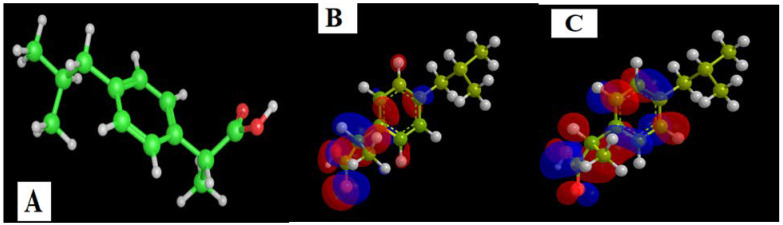
The ibuprofen drug’s DFT simulation. (**A**) 3D model, (**B**) HOMO and (**C**) LUMO.

The DFT was used to obtain representative data on the ibuprofen drug, as illustrated in Figure 7 and Table 6. The HOMO and LUMO orbitals provided information on the orbitals included in the excitation–relaxation process of electronic migration. The massive amount of occupied and unoccupied orbitals indicated that promoting electrons from level to level requires less energy. In Table 6, the HOMO, LUMO, and ΔE energies. The polarity of ibuprofen is shown by the dipole value (1.94 D), which is unfavorable to drug behavior [57].

##### Calculation of the Density Function for Hydrazide Ibuprofen (HI) 

The application of the DFT to HI produced the results shown in Figure S22 and mentioned in Table 6. The dipole value (3.37 D) matches the requirements of the new drug [58]. The HI ligand was equivalent to Ibuprofen, which has a high number of electrons and orbitals, indicating the potential to interact with the receptor and increase the drug’s value [57,59].

##### Calculation of the Density Function for HL Ligand

**Figure 8:**
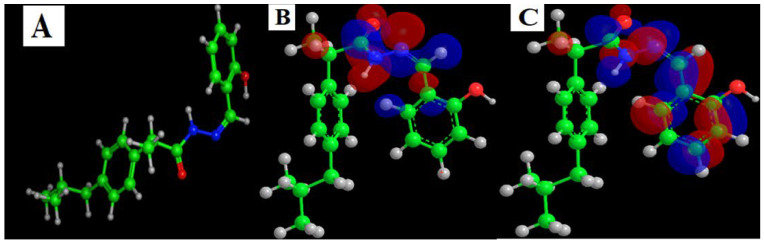
The HL Ligand is simulated using DFT. (**A**) 3D model, (**B**) HOMO and (**C**) LUMO.

Figure 8 and Table S4 illustrate the outcomes of DFT on the HL ligand. The dipole value (6.20 D) corresponds to the new drug’s requirements [58]. The HL ligand has atoms, orbitals, and electrons larger than the Ibuprofen drug, which indicates a change in their energy. The electron density map appeared to be more branched than the parent drug, adding other possibilities for receptor interaction and enhancing the drug’s potency by providing further favorable binding interactions.

As a result, the following has been shown:

1. The essential equations for defining the transition from the ground state to the next give a solid foundation for determining non-local, local, and global hardness and the role of softness;
2. The soft-soft and hard-hard interactions between two systems provide an optimum hardness;
3. It has been proven that the estimate system’s ground-state energy decreases as its hardness increases.

These broad assertions may be beneficial for analyzing reaction mechanisms and comprehending a molecule’s overall behavior when interacting with various chemicals [60].

### 2.4. Applications and Docking

#### 2.4.1. Docking Studies between Ibuprofen and Its Derivatives and COX2 (PDB Code: 5IKT)

Docking studies were used to model the interaction of ibuprofen, HI, HL, and the HL metal complexes with the target protein, COX2. These calculations provide insight into the energetics of drug candidate-COX2 interactions and which amino acid residues in COX2 interact with each drug candidate. 

##### Ibuprofen with COX2 Docking (PDB Code: 5IKT)

**Figure 9:**
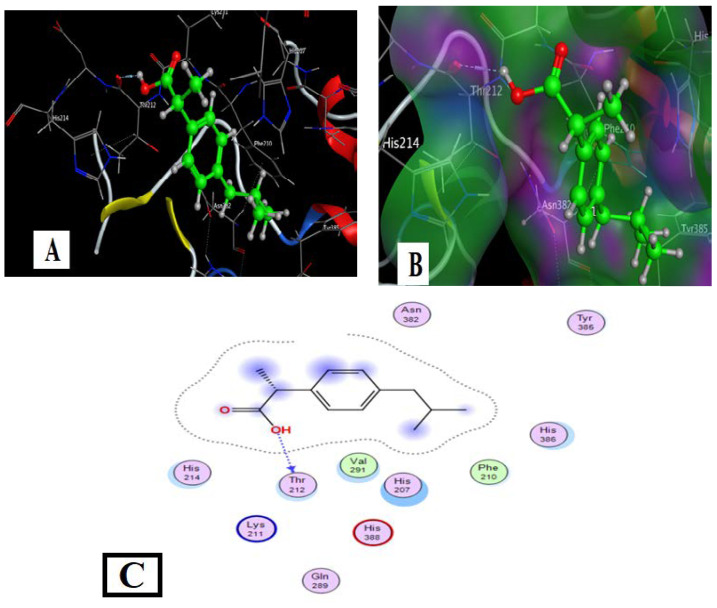
Docking model of Ibuprofen’s interaction with COX2 [PDB code: 5IKT] bonding sites: (**A**) 3D interaction diagram; (**B**) The surface properties [Hydrophilic sites (violet color), neutral sites (white color), and lipophilic sites (green color)]; and (**C**) 2D interaction diagram.

The drug’s molecular docking with COX2 is indicated in Figure 9 and Table 6. The data strongly suggested that the target ligand sites: O(14), interact with the target protein via the THR-212 amino acid. Hydrogen bonds and hydrophobic interactions (ex., Arene-H bonds) appeared as types of interaction bonds. The model with the most binding energy is shown in Figure 8. The binding energy was shown as −5.33 kcal/mol.

##### Docking of Hydrazide Ibuprofen (HI) with COX2 (PDB Code: 5IKT)

**Figure 10:**
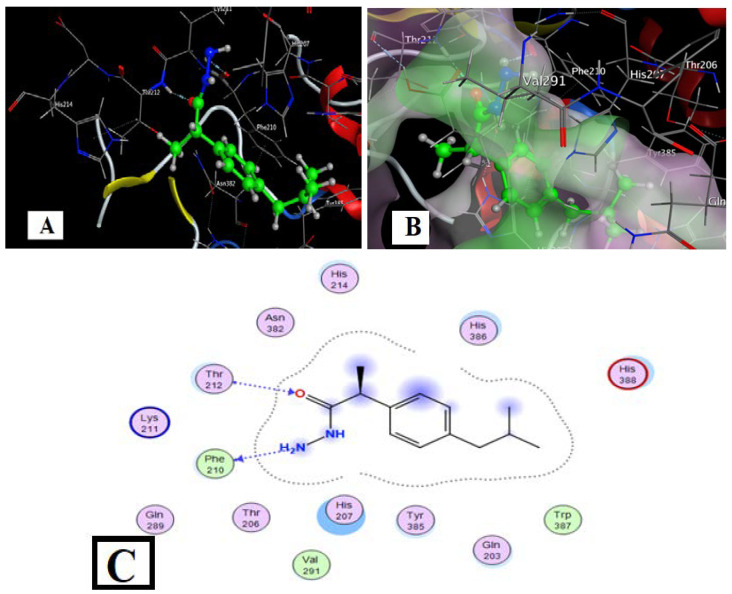
HI-COX2 interaction docking and model bonding sites: (**A**) 3D interaction diagram; (**B**) The surface properties [Hydrophilic sites (violet color), neutral sites (white color), and lipophilic sites (green color)]; and (**C**) 2D interaction diagram.

The docking of HI with COX2 was indicated in Figure 10 and Table 7. The chosen pose allowed the target ligand O(15) to interact with the target protein via an H-bond with THR-212, while N(16) created an arene-H bond with the PHE-210 amino acid. Hydrogen bonds and hydrophobic interactions (ex., Arene-H bonds) emerged as the most common interaction bonds, with the hydrogen bond type dominating. HI had binding energy of −5.65 kcal/mol, slightly greater than ibuprofen (Table 7).

##### Docking of HL Ligand with COX2 (PDB Code: 5IKT)

**Figure 11:**
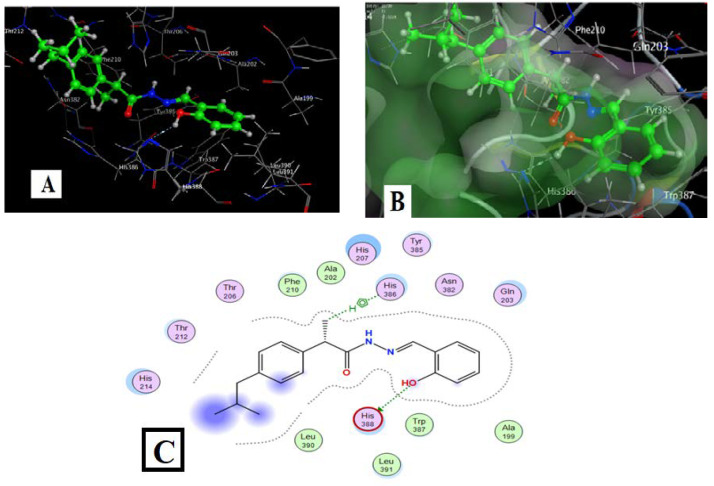
HL-COX2 interaction docking and model bonding sites: (**A**) 3D interaction diagram. (**B**) The surface properties [Hydrophilic sites (violet color), neutral sites (white color), and lipophilic sites (green color)]. (**C**) 2D interaction diagram.

Figure 11 and Table 8 show the docking results of Ligand HL with COX2. An H-bond led to the target ligand O(1) linkage with the HIS-388 amino acid of the target protein, and C(12) of the methyl group formed an arene-H bond with HIS 386 amino acid for the chosen position. Hydrogen bonds and hydrophobic interactions (ex., arene-H bonds) emerged as essential interaction bonds. HL had binding energy of −7.52 kcal/mol, which was more significant than ibuprofen (Table 6).

The binding energies of the compounds HI and HL ligands, which gave more negative binding energies than Ibuprofen, are summarized in Table 9. These more negative binding energies suggest that HI and HL are potential drug candidates whose biological properties should be studied in vitro.

#### 2.4.2. Molecular Docking of the Metal Complexes Derivatives from HL Ligand

##### Molecular Docking of the Co, Cu, Ni, Gd, and Sm Complexes

**Figure 12:**
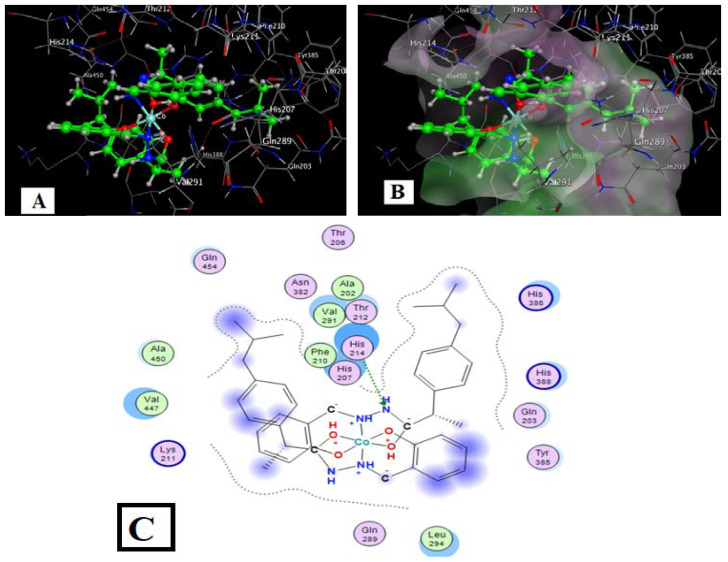
L-Co with COX2 interaction docking and model bonding sites: (**A**) 3D interaction diagram (**B**) The surface properties [Hydrophilic sites (violet color), neutral sites (white color), and lipophilic sites (green color)]. (**C**) 2D interaction diagram.

The docking data from the interaction between the metal complexes with COX2 (PDB Code: 5IKT) are listed in Table 10 and Figure 12 and Figures S23–S26.

The target complexes had more interaction sites than the free HL ligand and ibuprofen, while the Co complex showed more sites than ibuprofen but fewer than HL (Table 11). The total binding energies for the Co, Cu, Ni, Gd, and Sm complexes with COX were −9.41, −9.51, −8.09, −8.05, and −10.04 kcal/mol, respectively, which is somewhat higher than the ligand HL (−7.52 kcal/mol) and higher than the ibuprofen drug (−5.33 kcal/mol). These increased binding energies suggest that the metal complexes derived from HL should be studied in biochemical and cell studies in vitro.

### 2.5. In Silico Physicochemical Descriptors, Pharmacokinetic Properties, and Bioactivity Prediction

Drug-likeness is a delicate balancing act of several molecular characteristics and structural properties. Bioavailability, transport characteristics, affinity to proteins, reactivity, and many other properties influence the behavior of a molecule in a live body.

The chemical structures of the ibuprofen drug, synthesized compound HL, and its cobalt complex were incorporated in the web application SwissADME to discover substructure characteristics, which in turn dictate physicochemical parameters [61].

Physicochemical properties such as the heavy atoms number, rotatable bonds, H-bond acceptors, H-bond donors, the fraction of carbon bond saturation (Csp^3^), i.e., the number of sp^3^ hybridized carbons/total carbon count, topological polar surface area (TPSA), molar refractivity, water solubility (S) parameter LogS (Silicos-IT), and lipophilicity parameter LogP using the additive XLogP3 approach to hypothesize [62] and the Wildman–Crippen method (WLogP) [63].

For each compound, pharmacokinetic factors such as Blood-Brain Barrier permeation (BBB permeation), gastrointestinal absorption (GI absorption), P-glycoprotein substrate (P-gp substrate), and skin permeation (Log Kp) were calculated and were listed in Table 11. 

**Table 11:** Predicted physicochemical descriptors of ibuprofen drug, the synthesized compounds HL, and its cobalt complexes.

Compound	Molecular Weight (g/mol)	Heavy Atoms	Rotatable Bonds	H-Bond Donors	H-Bond Acceptors	Fraction Csp3	Solubility LogS (Silicos-IT) (Water)	XLogP3	WLOGP	Molar Refractivity	TPSA (Å^2^)	GI Absorption	BBB Permeant	P-gp substrate	Log Kp (cm/s)
Ibuprofen	206.28	15	4	1	2	0.46	−3.44	3.50	3.07	62.18	37.30	High	Yes	No	−5.07
HL	324.42	24	7	2	3	0.30	−6.07	5.47	3.84	98.41	61.69	High	Yes	No	−4.40
L-Co	723.77	50	8	1	7	0.35	−11.8	11.17	7.29	203.71	77.35	High	No	Yes	−2.78

The Brain or Intestinal Estimated Permeation approach was employed to estimate and conclude brain penetration (yes or no), gastrointestinal absorption (High or Low), and P-glycoprotein substrate (yes or no) (BOILED-Egg model) [64,65].

**Figure 13:**
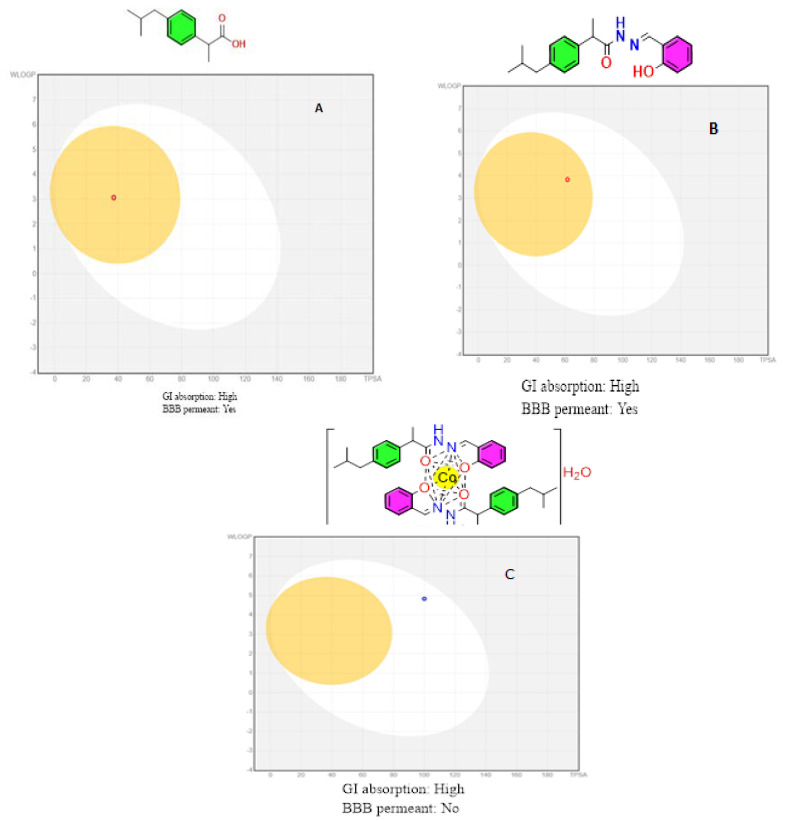
BOILED-Egg model for: (**A**) Ibuprofen drug; (**B**) Ligand HL; and (**C**) L-Co complex.

The white zone indicates the predictability of slow absorption through the gastrointestinal system, while the yellow region (yolk) indicates a significant likelihood of brain penetration. The yolk and white parts are not necessarily incompatible. The points are also colored blue if they are expected to be actively effluxed by P-glycoprotein (PGP+) and red if they are expected to be non-substrate of P-glycoprotein (PGP) (Figure 13). This model is based on two factors: (1) the lipophilicity of the compounds under investigation, expressed as a partition coefficient (P) using the Wildman–Crippen method (WLogP) [63], and (2) The polarity of the compounds, expressed as a topological polar surface area (TPSA) value.

Points in the BOILED-Egg’s yolk is estimated to passively permeate through the BBB, while points in the BOILED-Egg’s white are estimated to be passively absorbed via the GI tract in this model. The Predicted pharmacokinetic properties of ibuprofen and HL ligand showed a high absorption by GIT while passively permeable through the BBB except for the Co complex (Figure 13).

The developed compounds were evaluated according to Veber’s rule-based method [66,67] to determine their drug-likeness. When there are ten or fewer rotatable bonds and a polar surface area equal to or less than 140 Å^2^, excellent bioavailability is expected. From the data provided in Table 11, compounds HL and the Co complex showed zero violations and can be considered promising drug candidates for bioactivity studies.

The partition coefficient value strongly reflects the ionic-covalent behavior of the target compounds. Based on the Wildman–Crippen method (WLogP), the lipophilicity of the ibuprofen drug, the synthesized compounds HL, and the cobalt complex were measured as a partition-coefficient (P) [63].

The compounds HL and L-Co (3.84 and 7.29) have higher n-octanol/water partition coefficients than ibuprofen (3.07), as shown in Table 11, indicating high lipophilicity. The high GI absorptivities predicted for HL and L-Co suggest that they may be bioavailable following oral administration, and that in vivo studies in mice are worthwhile.

### 2.6. In Vitro Anti-Inflammatory Activity

#### 2.6.1. Cyclooxygenase Inhibition Assay

The in vitro COX-1/COX-2 isozyme inhibition assays determined the ability of compounds HI and HL to inhibit ovine COX-1 and human recombinant COX-2 using an enzyme immunoassay (EIA). In addition, the COX-2 selectivity indices [SI values = IC_50_ (COX-1)/IC_50_(COX-2)] were estimated and compared with that of ibuprofen, indomethacin, and diclofenac sodium as standard drugs (Table 12 and Table S5). The results revealed that both compounds (HI and HL) had a wide range of COX-1 (IC_50_ = 7.5 and 8.6 µM), and COX-2 (IC_50_ = 4.3 and 4.9 µM) inhibitory activities. HI and HL showed higher COX-2 selectivity indices (SI = 1.7 and 1.8) compared to ibuprofen, indomethacin and diclofenac sodium which had COX-2 selectivity indices (SI = 0.4, 5.0 and 4.5, respectively).

The complexes of HL were tested as predicted COX-2 inhibitors (Table S5), where cobalt complex was the most active (IC_50_ = 1.7 µM). Complexes of copper, nickel, gadolinium, and samarium had IC_50_ of 5.6, 3.7, 2.3 and 2.9 µM, respectively.

All derivatives were more potent inhibitors of COX-1/COX-2 than ibuprofen.

#### 2.6.2. Western Blot Analysis

**Figure 14:**
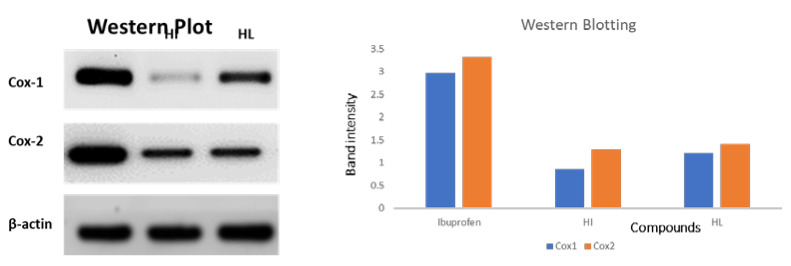
Western blot of the synthesized compounds against both COX-1 and COX-2 enzymes.

The anti-inflammatory activities of compounds HI and HL were further confirmed by Western blotting analysis of COX-1/COX-2. The compounds induced a remarkable DNA fragmentation and downregulation of COX-1 and COX-2 than the untreated control. These results followed the cyclooxygenase inhibition assay results (Figure 14).

#### 2.6.3. Cell Viability Using MTT Assay

**Figure 15:**
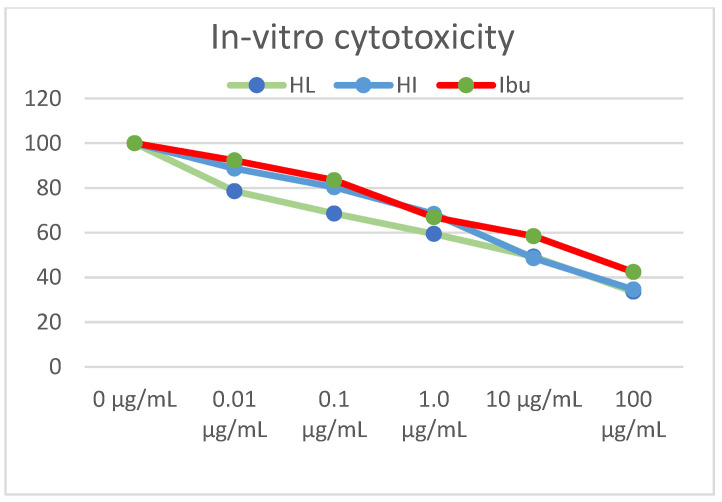
In-vitro cell viability assay of synthesized compounds on the fibroblasts cell line. At 5 × 104 cells/well cell density, cells were treated with several drug concentrations (0.1, 1, 10, and 100 µM) of investigated compounds or positive comparator, ibuprofen, for 24 h exposure time. Cytotoxicity was identified via MTT-based colorimetric assay (450 nm), and % cell growth viability was estimated in triplicates, showing HI and HL as active derivatives relative to ibuprofen as standard control.

The synthesized compounds were screened for their cytotoxic activities against fibroblast cell lines at different concentrations for 24 h using MTT assay. The results illustrated in Table 13 and Figure 15 showed that compounds HI and HL had a promising COX-2 inhibitory effect with IC_50_ values of 3.90 and 4.13 µM, respectively, compared to ibuprofen (IC_50_ = 30.60 µM).

### 2.7. DNA Interaction

**Figure 16:**
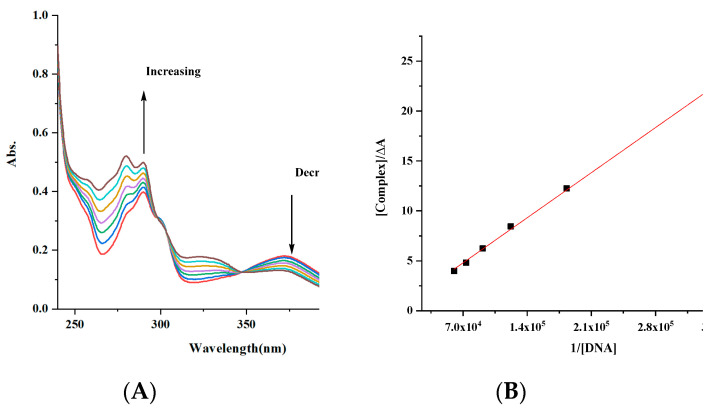
(**A**) Electronic spectra of complex [complex (Gd-L1)] = 6.02 × 10^−5^ M with increasing the concentration of DNA [DNA] = 2.73 × 10^−6^–1.64 × 10^−5^ M; (**B**) Benesi–Hildebrand fitting curve. The arrows show the intensity changes upon increasing concentrations of the DNA.

**Figure 17:**
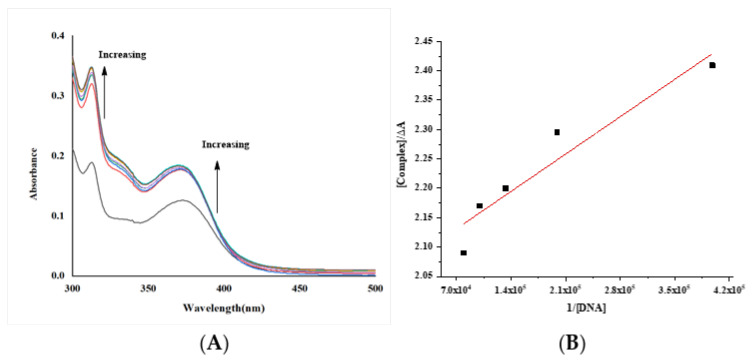
(**A**) Electronic spectra of complex [complex (Cu-L1)] = 1.9 × 10^–5^ M with increasing the concentration of DNA [DNA] = 2.51 × 10^−6^ – 1.76 × 10^−5^ M; (**B**) Benesi–Hildebrand fitting curve. The arrows show the intensity changes upon increasing concentrations of the DNA.

**Figure 18:**
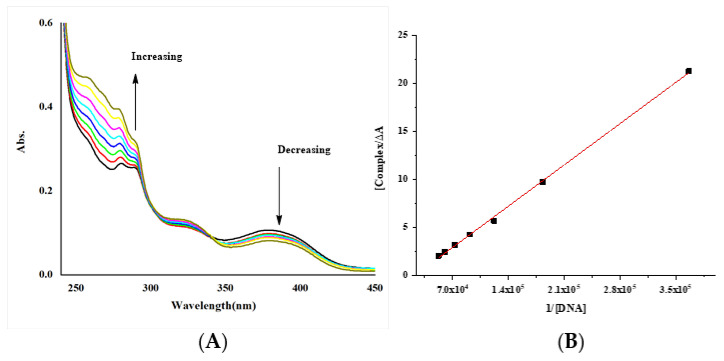
(**A**) Electronic spectra of complex [complex (Co-L1)] = 4.04 × 10^−5^ M with increasing the concentration of DNA [DNA] = 2.73 × 10^−6^ – 1.64 × 10^−5^ M; (**B**) Benesi–Hildebrand fitting curve. The arrows show the intensity changes upon increasing concentrations of the DNA.

The absorption spectra of the Co, Cu and Gd complexes in the absence and presence of DNA are shown in Figure 16, Figure 17 and Figure 18. With increasing concentrations of DNA, the absorption bands of the complex were influenced, resulting in the pattern of bathochromic of the Cu complex due to the strong stalking interaction of the complex and the base pairs of DNA. The Co and Gd complexes give a very slight blue shift.

So, the observed hyperchromic reflects structural damage, likely due to the complexes’ strong binding to the DNA base moieties through covalent-bond formation [55,68,69,70].

The intrinsic binding constants K_b_ of the complexes were 4.46 × 10^3^, 2.2 × 10^4^ and 2.27 × 10^6^ M^−1^ for the Gd, Co and Cu complexes. The obtained values showed that the complexes are moderately bound to DNA. However, the value of the Cu complex is like those of classical intercalators (K_b_ × 10^7^ M^−1^) [58,70,71].

## 3. Materials and Methods

### 3.1. Materials

All the chemicals are analytical-grade reagents. Ibuprofen in its purest form (Sigma Aldrich, Hamburg, Germany, cat# I110), salicylaldehyde, copper(II) chloride (CuCl_2_), nickel(II) chloride (NiCl_2_), cobalt(II) chloride hexahydrate (CoCl_2_·6H_2_O), gadolinium(III) nitrate hexahydrate (Gd(NO_3_)_3_·6H_2_O), samarium(III) nitrate hexahydrate (Sm(NO_3_)_3_·6H_2_O), HPLC-grade solvents, Tris HCl buffer and highly polymerized fish-milt DNA (FM-DNA) were obtained from Sigma-Aldrich. All glassware and other equipment were cleaned with distilled water and dried in the oven before usage.

### 3.2. Instrumentation

The melting points in open capillaries were determined using electrical melting point equipment. A WTW digital conductivity meter, Xylem Analytics, Weilheim, Germany measured the complexes’ electrical conductivity at ambient temperature on 10^−3^ M DMSO solutions. Microanalyses of carbon, hydrogen, and nitrogen were carried out using a CHNS-932 (LECO) elemental analyzer, Hunan Sundy Science and Technology Co., Ltd., Hunan, China. Analyses of the metals were conducted by dissolving the solid complexes in concentrated HNO_3_/H_2_O_2_ and dissolving the residue in deionized water. The Co, Ni, Cu, Sm and Gd metal content was measured using EDTA titration with a murexide indicator in ammonia buffer (pH 10) or thermogravimetric analysis. The ^1^H NMR spectrum was registered on Varian spectrometer using DMSO-d_6_ as a solvent and tetramethylsilane (TMS) as a standard, with chemical shifts given in ppm. The electronic spectra of the metal complexes were recorded on a UV-1800 Shimadzu spectrophotometer (800–200 nm) using a quartz cuvette (1 cm path length), Shimadzu, Nakagyo, Kyoto, Japan. The electronic absorption spectra of solid complexes in the Nujol mull were calculated using Lee et al. [72]. The FTIR spectra of the Schiff bases and their complexes were obtained on a Bruker Tensor 27 spectrophotometer (KBr disc) in the 400–4000 cm^−1^ range, Bruker Corporation, Billerica, Massachusetts, USA. A Shimadzu Qp-2010 plus mass analyzer was registered for mass spectrometry Shimadzu, Nakagyo, Kyoto, Japan. Mercury(II) tetrathiocyanatocobaltate(II) was used as a standard for magnetic susceptibility measurements on the MSB-MK1 balance at ambient temperature using the modified Gouy method(II). Under a nitrogen flow (20 mL/min) and 10 °C/min heating rate in the 40–800 °C range, thermal analysis (TGA, DTG, and DTA) was performed on a Shimadzu 60H thermal analyzer, Shimadzu, Nakagyo, Kyoto, Japan. The molecular modeling of the produced compounds was done using the Chem Office software program (Version 16.0) to perform an energy optimization operation using the MM2 computation.

### 3.3. Synthesis

For synthesizing ester and hydrazine, we used the conventional method widely used in the literature [73,74,75], with some deviation in the reflux time.

#### 3.3.1. Ethyl 2-(4-Isobutyl Phenyl) Propanoate (IE) Synthesis

Ibuprofen (10 g) was dissolved in 40 mL of absolute ethanol, then four drops of concentrated H_2_SO_4_ were added, and the mixture was heated at reflux for 16 h with continuous stirring. Thin-layer chromatography (TLC) was utilized to assess reaction completion. The reaction mixture has added a mixture of cold distilled water and 5% Na_2_CO_3_. The separating funnel separated the aqueous solution from the organic solution (ethyl Ibuprofen) after 24 h.

#### 3.3.2. 2-(4-Isobutylphenyl) Propane Hydrazide (HI) Synthesis

IE (C_15_H_22_O_2_) (6 g) was dissolved in 30 mL absolute ethanol, and then 9 mL 80% NH_2_NH_2_ in H_2_O was gradually added while stirring. The combination was refluxed for 16 h. TLC confirmed the end of the reaction. The mixture was evaporated to a third of its original volume, set aside to cool at ambient temperature, and the residue was filtered to obtain the product. Then, the product was recrystallized from hot methanol and dried under a vacuum over anhydrous CaCl_2_ forming pure white crystals with a melting point of 85 °C.

#### 3.3.3. Ibuprofen and Salicylaldehyde Hydrazone (HL) Synthesis

**Scheme 3:**
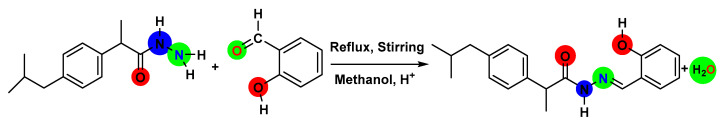
The Schiff base HL synthesis.

Ibuprofen hydrazide (C_13_H_20_N_2_O) (1 mmol, 0.22 g, in 30 mL methanol) and salicylaldehyde (1 mmol, 0.11 mL) were mixed by dropwise addition. The resulting mixture was heated at reflux for 3 h to completeness as estimated by TLC. Evaporation decreased the solution volume to one-fifth of its initial volume and then left to cool at ambient temperature. The canary-yellow precipitate was filtered, rinsed with methanol, and dried in a desiccator with anhydrous CaCl_2_ (Scheme 3).

#### 3.3.4. Metal Complex Synthesis

The stable metal complexes were formed by dropwise addition of a hot ethanolic solution of 0.1 mmol of CuCl_2_ (0.067 g), NiCl_2_ (0.0648 g), CoCl_2_·6H_2_O (0.1189 g), Sm(NO_3_)_3_·6H_2_O (0.222 g), or Gd (NO_3_)_3_·6H_2_O (0.225 g) with continuous stirring to a hot ethanolic solution of 0.2 mmol of HL (0.3245 g). The solution was refluxed by constantly stirring for 2–3 h and standing at room temperature. The mixture pH was adjusted to 7–8 for Co, Ni, and Co complexes, while in the case of Gd and Sm complexes, to 4–5.5. By reducing the amount of the solution, different colored solid complexes were produced. The precipitate was filtered to a solid crystal, washed with methanol, and dried in desiccators.

### 3.4. In Vitro Anti-Inflammatory Activity

#### 3.4.1. Enzyme-Linked Immunosorbent Assay (ELISA)

The ability of hydrazide, HL, and some complexes to inhibit bovine COX1 and human recombinant COX2 (based on their IC_50_ values) was confirmed using an enzyme-linked immunosorbent assay (ELISA) kit (item no. 560131, Cayman Chemical, Ann Arbor, MI, USA) based on the protocols established by Cayman Chemical [76,77].

#### 3.4.2. Western Blot Analysis

For validating the anti-inflammatory pathway at the protein expression level, we used the immunoblotting (Western blot) technique [78,79].

#### 3.4.3. Cell Viability Using MTT Assay

Human Dermal Fibroblast (HDF) cell line was bought from the American Type Culture Collection (ATCC; Minnesota, USAHB-8065). It was cultured in Park Memorial Institute (RPMI-1640) culture media with glutamine 2 mM (bio west, Nampa, cat. no. L0498-500), 10% fetal bovine serum (PAA, Pasching Austria, cat. no. A11-151) and penicillin with streptomycin 1% (Lonza, Verviers, Belgium, cat. no. DE17-602E). The HDF cells were grown in 50 cm^2^ flask (Greinerbio-one GmbH Maybach-str. 272636 Frickenhausen, Germany) and preserved in a typical humidified incubator supplied with 5% CO_2_, 95% air at 37 °C (New Brunswick Scientific-Innova co-170). Cells were washed with cold phosphate-buffered saline, trypsinized, harvested and centrifuged to form cell pellets. The cultured HDFa were plated in 96 well microplates at a concentration of 5 × 104 cells per well 24 h before the MTT assay to allow microplate adherence. MTT Reagent was provided, prepared for use, and acquired from Biospes, China, Cat n#BAR1005-1. According to the manufacturer protocol [80,81,82], the cultured cells were presented with different concentrations of the assessed agents. 20 μL of MTT solution was filled with each well. For 4 h, the plate was kept at 37 °C. After incubation, 100 μL of formazan diluent buffer (Biospes, Chongqing, China, Cat n#BAR1005-1) was poured into each well. ELISA plate reader (Stat Fax 2200, Awareness Technologies, Palm City, FL, USA) measured color absorbance at 540 nm [83]. Three readings were taken on three different days and averaged for each sample. At last, cell survival was determined according to the following equation:

$$\%\mathrm{cell}\;\mathrm{survival}=\frac{A_{\mathrm{sample}}}{A_{\mathrm{control}}}\times 100\%$$

A_control_ is the absorbance of the cell culture after adding MTT and formazan buffer, but without treatment by any drug candidate. At the same time, A_sample_ is the corresponding absorption for samples treated with the drug candidates. IC_50_ values were then calculated by curve-fitting involving non-linear regression [84].

### 3.5. DNA Interaction

#### Ultraviolet-Visible Absorption Spectroscopy

The DNA concentration was estimated using absorbance at 260 nm (=6600 cm^−1^). The test was performed in a pH 7.4 Tris-HCl buffer [85]. The ratio of absorption at 260 nm and 280 nm was determined to be 1.87, indicating that there was no protein contamination with DNA [86]. The DNA solution was stored at a temperature of 4 °C. Doubly distilled water was used to produce all the working solutions.

In a methanolic solution, the electronic absorption spectra of the formed complexes were measured in the presence and absence of DNA. At 25 °C, these titrations were carried out by maintaining the complex concentration fixed (1.9–6.02 × 10−5 M) and altering the DNA concentration (2.51 × 10^−6^–1.91 × 10−5 M). We added the same amount of DNA to the sample and the reference with each addition to account for absorption by DNA.

## 4. Conclusions

The novel ibuprofen hydrazine and salicylaldehyde hydrazone (HL) and its metallo-derivatives with Cu(II), Ni(II), Co(II), Gd(III), and Sm(III) were prepared and characterized using ^1^HNMR, UV-visible, and FT-infrared spectroscopy, thermal analysis (DTA and TGA), conductivity, magnetic susceptibility, and elemental analysis. The results indicated the formation of the novel complexes [Cu(L)(H_2_O)]Cl.2H_2_O, [Ni(L)_2_], [Co(L)_2_].H_2_O, [Gd(L)_2_(H_2_O)_2_].(NO_3_).2H_2_O and [Sm(L)_2_(H_2_O)_2_](NO_3_).2H_2_O complexes. Density function. The theory was used to calculate the surface properties of the prepared compounds. Docking analysis calculations with COX2 as the target were consistent with the in vitro studies. Generally, computational modeling suggests that HL and its complexes should display higher bioactivity than ibuprofen. The BOILED-Egg model suggested that HL and its Co complex will have high gastrointestinal absorption, while only HL has the ability to penetrate the blood-brain barrier. Cyclooxygenase inhibition assays showed that the novel ibuprofen derivatives have lower IC_50_ values for the inhibition of COX1 and COX2 than ibuprofen itself. The metal complexes were tested for interaction with DNA, and these experiments showed strong interactions with the intercalating binding type. These results suggest that the new prepared compounds should be studied in animal models as potential anti-inflammatory drugs.

## Figures and Tables

**Table 1:** Conductivity measurements and analytical results of HL Schiff base and its complexes.

Compound	Molecular Weight	Color	Melting Point (°C)	Ω * (µS)	CHNM%					
					C%	H%	N%	M% **		
					Calc. Found	Calc. Found	Calc. Found	A	B	Calc.
HL(C_20_H_24_N_2_ O_2_)	324.42	Canary-yellow	159	---	74.05 74.10	7.46 7.51	8.63 8.55	---	---	---
[Cu(L)(H_2_O)]Cl.H_2_O	458.48	Dirty green	>280	40	52.40 52.42	5.94 5.32	6.11 5.92	13.23	13.71	13.86
[Ni(L)_2_]	705.53	Apple green	>280	8	68.10 68.22	6.57 6.62	7.94 7.89	8.20	8.09	8.32
[Co(L)_2_]1/2H_2_O	714.78	Brick brown	>280	10	66.36 66.42	6.72 6.73	7.83 7.72	8.03	8.24	8.14
[Gd(L)_2_(H_2_O)_2_](NO_3_).3/2H_2_O	929.16	Buff	>280	100	51.66 50.35	5.38 5.89	7.52 7.42	---	16.16	16.45
[Sm(L)_2_(H_2_O)_2_](NO_3_).2H_2_O	931.25	Yellow-orange	>280	108	51.55 51.60	5.80 5.85	7.52 7.48	---	16.03	16.14

* 10^−3^ M in DMSO, ohm^−1^ cm^2^ mol^−1^. ** A = complexometric titration and B = thermo-gravimetric analysis.

**Table 3:** Complexes formed from HL Schiff base TGA and DTG.

Compound	Temp. Range °C	DTG Temp. °C	Mass Loss %		Operation	Expected Products	Residue	M %
			Found	Calcd.			(Calcd.) Found	
[Cu(L)(H_2_O)]Cl·H_2_O	22–110	69	3.68	3.93	Dehydration	H_2_O	CuO (16.71) 17.1	13.17
	120–350	250	46.62	46.45	Coordination sphere + ligand decomposition	H_2_O + HCl + 0.48 L		
	350–800	606	32.8	32.66	Final decomposition	0.46 L		
[Ni(L)_2_]	50–291	234	15.35	15.67	ligand decomposition	0.17 L	NiO (10.55) 10.30	8.09
	300–396	338, 371	46.25	46.21	ligand decomposition	0.51 L		
	398–800	449	28.4	28.26	Final decomposition	0.31 L		
[Co(L)_2_]·1/2H_2_O	51–170	64	1.25	1.31	Dehydration	1/2H_2_O	CoO (10.9) 10.62	8.24
	319–358	337	40.58	40.71	ligand decomposition	0.43 L		
	453–800	473	46.63	46.39	Final decomposition	0.49 L		
[Gd(L)_2_(H_2_O)_2_](NO_3_)·3/2H_2_O	24–134	73	2.83	2.91	Dehydration	3/2H_2_O	GdO 0.17 L ligand residue (30.88) 29.47	16.16
	134–244	190	3.9	3.76	Coordination sphere	2H_2_O		
	250–448	330, 405	32.1	32.5	NO_3_ liberation + ligand decomposition	HNO_3_ + 0.37 L		
	452–761	510, 630	29.7	30.03	Final decomposition	0.43 L		
[Sm(L)_2_(H_2_O)_2_](NO_3_) ·2H_2_O	30–171	83	3.65	3.87	Dehydration	2H_2_O	SmO 0.11 ligand residue (23.81) 24.31	16.03
	174–338	329	27.49	27.56	Coordination sphere + ligand decomposition	2H_2_O + 0.34 L		
	438–477	441	23.37	23.38	NO_3_ liberation + ligand decomposition	HNO_3_ + 0.24 L		
	482–492	499	21.21	21.58	Final decomposition	0.31 L		

**Table 4:** The specific degradation steps of the HL ligand complexes’ kinetic parameters.

Complex	Step	R^2^	Order (n)	Ts (K)	ΔEa (J/mol)	Z (s^−1^)	ΔS* (J/K.mol)	ΔH* (kJ/mol)	ΔG* (kJ/mol)
[Cu(L)(H_2_O)]Cl.2H_2_O	Final ligand Degradation	0.95	2.00	876	184.05	6.41 × 10^10^	−46.99	−6.849	34.31
[Ni(L)_2_]	Final ligand Degradation	0.95	2.00	721	74.83	1.07 × 10^5^	−156.00	−5.919	63.97
[Co(L)_2_]1/2H_2_O	Final ligand Degradation	0.99	2.00	745	104.97	1.31 × 10^7^	−116.29	−6.089	80.55
[Gd(L)_2_(H_2_O)_2_](NO_3_).3H_2_O	ligand degradation	0.97	0.66	608	102.48	5.22 × 10^6^	−122.26	−4.952	69.38
[Sm(L)_2_(H_2_O)_2_](NO_3_).3/2H_2_O	NO_3_ liberation + ligand degradation	0.99	2.00	721	136.10	2.24 × 10^10^	−54.12	−5.858	33.16

**Table 5:** The synthesized chemicals’ surface characteristics.

Ligand	Active Lone Pair Map	Lipophilic and Hydrophilic Map
HI	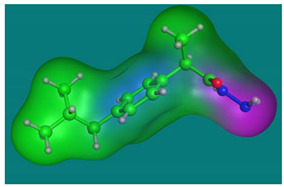	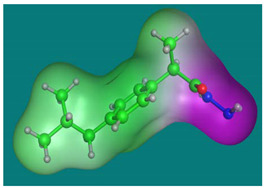
HL	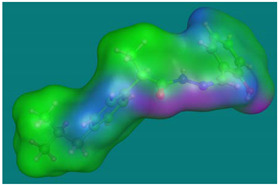	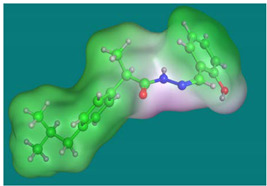

**Table 6:** The Ibuprofen’s interaction parameters with COX2 [PDB code: 5IKT].

Ibuprofen	Ligand Sites	Receptor Sites	Type of the Interaction	Bond Distance (Å)	Binding Energy (kcal/mol)	The Total Free-Binding Energy (kcal/mol)
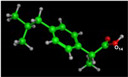	O(14)	THR 212	H-donor	2.85	−2.9	−5.33

**Table 7:** The parameters of the HI-COX2 interaction.

Ligand	Ligand Sites	Receptor Sites	Type of the Interaction	Distance of Bond (Å)	Binding Energy (kcal/mol)	Total Free Binding Energy (kcal/mol)
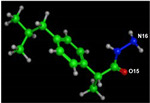	O(15)	THR 212	H-accept or H-donor	3.05	−2.7	−5.65
	N(16)	PHE 210		3.01	−1.9	

**Table 8:** The parameters of the HL-COX2 interaction.

Ligand	Ligand Sites	Receptor Sites	Interaction Type	Distance (Å)	Energy of Binding (kcal/mol)	The Energy of Total Free Binding (kcal/mol)
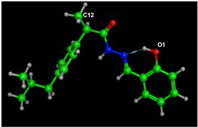	O(1)	HIS 388	H- donor	2.87	−1	−7.52
	C(12)	HIS 386	H-pi	4.34	−0.8	

**Table 9:** Computational binding energies of the synthesized compounds.

Compound	PDB 5IKT (Homo Sapiens) S-Value (Kcal/mol)
Ibuprofen	−5.33
HI	−5.65
HL	−7.52

**Table 10:** The metal complexes derived from HL with COX2 [PDB code: 5IKT] observed interaction parameters.

Compound	Sites of Ligand	Receptor Sites	Interaction Kind	Bond Distance (Å)	Binding Energy (kcal/mol)	The Energy of Total Free Binding (kcal/mol)
HL	O(1)	HIS 388	H-donor	2.87	−1	−7.52
	C(12)	HIS 386	H-pi	4.34	−0.8	
Co-L	N(11)	HIS 214	H-acceptor	2.97	−1.12	−9.41
Cu-L	O(1)	HIS 388	H-donor	2.97	−4.6	−8.09
	Ph	HIS 386	H-pi	4.39	−1.6	
Ni-L	Ph	HIS 214	H-pi	3.55	−0.8	−9.51
	Ph	HIS 386	H-pi	4.19	−0.6	
Gd-L	N(10)	HIS 214	H-acceptor	3.18	−2.3	−8.05
	Ph	LYS 211	H-pi	4.15	−1	
	Ph	HIS 386	pi-pi	3.94	0	
Sm-L	Ph	HIS 386	H-pi	4.28	−0.8	−10.04
	Ph	HIS 217	H-pi	3.79	−0.6	
	Ph	GLN 203	H-pi	4.47	−0.6	

**Table 12:** In vitro COX-1 and COX-2 inhibition of HI and HL.

Compound	IC_50_ (µM) ^a^		
	COX-1	COX-2	COX-2 SI ^b^
HI	7.5	4.3	1.7
HL	8.6	4.9	1.8
Ibuprofen	12.9	31.4	0.4
Indomethacin	0.4	0.1	5.0
Diclofenac sodium	3.8	0.8	4.5

^a^ IC_50_ value represents the compound concentration that is required to produce 50% inhibition of COX-1 or COX-2 which is the mean value of three determinations where the deviation from the mean is <10% of the mean value. ^b^ Selectivity index (COX-1 IC_50_/COX-2 IC_50_).

**Table 13:** Summary of IC_50_ (µM) values of compounds HI and HL against fibroblast cells, IC_50_ values are calculated through EXCEL using non-linear regression curve fit of sigmoidal dose-response inhibition.

Compound No.	Fibroblast Cells
HL	4.13
HI	3.9
Ibuprofen	30.6

## Data Availability

All data generated or analyzed during this study are included in this published article (and its Supplementary Information Files).

## Supplementary Materials

The following supporting information can be downloaded at: https://www.mdpi.com/article/10.3390/molecules27217540/s1, Table S1: The ^1^H NMR data of the HI and HL; Table S2: DTA of Complexes derived from HL Schiff base; Table S3: The electronic spectra and magnetic features of the HL ligand and its complexes; Table S4: The data from DFT calculations and the properties of synthesized compounds; Table S5: In vitro COX-1 and COX-2 inhibition of the synthesized derivatives; Figure S1: ^1^HNMR spectrum of Hydrazide Ibuprofen (HI); Figure S2: Chromatogram and mass spectrum of the Ibuprofen hydrazone (HL) and hydrazide (HI); Figure S3: UV-Vis. Spectrum of the ligand HL; Figure S4: Thermal decomposition of Schiff base HL; Figure S5: FTIR spectrum of [Cu(L)(H_2_O)]Cl.2H_2_O complex; Figure S6: FTIR spectrum of [Ni(L)_2_] complex; Figure S7: FTIR spectrum of [Co(L)_2_]H_2_O complex; Figure S8: FTIR spectrum of [Gd(L)_2_(H_2_O)_2_] (NO_3_).3H_2_O complex; Figure S9: FTIR spectrum of [Sm(L)_2_(H_2_O)_2_] (NO_3_).2H_2_O complex; Figure S10: TGA/TG and DTA curves of [Cu(L)(H_2_O)]Cl.H_2_O complex; Figure S11: TGA/TG and DTA curves of [Ni(L)_2_] complex; Figure S12: TGA/TG and DTA curves of [Co(L)_2_]1/2H_2_O complex; Figure S13: TGA/TG and DTA curves of [Sm(L1)_2_(H_2_O)_2_](NO_3_).2H_2_O complex; Figure S14: The fit-linear curve of the liberation of coordinate water step of Cu-L1 complex; Figure S15: Fit-linear curve of liberation of coordinate water step of Ni-L1 complex; Figure S16: Fit-linear curve of liberation of coordinate water step of Co-L1 complex; Figure S17: Fit-linear curve of liberation of coordinate water step of Gd-L1 complex; Figure S18: Fit-linear curve of liberation of coordinate water step of Sm-L1 complex; Figure S19: UV-Vis. spectrum of L1-Ni; Figure S20: UV-Vis. spectrum of L1-Co; Figure S21: UV-Vis. spectrum of L1-Sm; Figure S22: The DFT simulation for the Hydrazide Ibuprofen. (A) 3D view, (B) HOMO and (C) LUMO; Figure S23: Docking model of the interaction of Cu-L1 with COX2 [PDB code: 5IKT] bonding sites: (A)3D interaction diagram (B) The surface properties [Hydrophilic sites (violet color), neutral sites (white color) and lipophilic sites (green color)]. (C) 2D interaction diagram; Figure S24: Docking model of the interaction of Ni-L1 with COX2 [PDB code: 5IKT] bonding sites: (A) 3D interaction diagram (B) The surface properties [Hydrophilic sites (violet color), neutral sites (white color) and lipophilic sites (green color)]. (C) 2D interaction diagram; Figure S25: Docking model of the interaction of Gd-L1 with COX2 [PDB code: 5IKT] bonding sites: (A) 3D interaction diagram (B) The surface properties [Hydrophilic sites (violet color), neutral sites (white color) and lipophilic sites (green color)]. (C) 2D interaction diagram; Figure S26: Docking model of the interaction of Sm-L1 with COX2 [PDB code: 5IKT] bonding sites: (A) 3D interaction diagram (B) The surface properties [Hydrophilic sites (violet color), neutral sites (white color) and lipophilic sites (green color)]. (C) 2D interaction diagram; Scheme S1: The chromatogram of pathway fragmentation of Hydrazide Ibuprofen (HI); Scheme S2: The chromatogram of pathway fragmentation of HL ligand.
