# Supplementary material for: A Novel Ibuprofen Derivative and Its Complexes: Physicochemical Characterization, DFT Modeling, Docking, In Vitro Anti-Inflammatory Studies, and DNA Interaction
Source: Molecules. 2022 Nov 3;27(21):7540. doi: 10.3390/molecules27217540 (PMC9653649; doi:10.3390/molecules27217540)
Supplement: Supplementary file 1 [file molecules-27-07540-s001.zip › molecules-1948225-supplementary.pdf]

A Novel Ibuprofen Derivative and Its Complexes: Physicochemical Characterization, DFT Modeling, Docking, In Vitro Anti-Inflammatory Studies, and DNA Interaction

Abbas M. Abbas [1,*] , Ahmed Aboelmagd [1], Safaa M. Kishk [2] , Hossam H. Nasrallah [1,3] , Warren Christopher Boyd [4], Haitham Kalil [1,4,*] and Adel S. Orabi [1,*]

Table S1: The ^{1}H NMR data of the HI and HL.

Comp.	δ (ppm)	
**HI**	9.13(1H, br, NH), 7.21-7.04 (4H, m, ArH), 4.18(2H, s, NH_2), 3.45(2H, s, CH_2), 2.39 (2H, s, CH_2), 1.80(1H, s, CH), 1.29 (3H, m, CH_3), 0.84-0.82(6H, m, $(CH_3)_2$).	
	**Δ (ppm) map (experimental)**	**δ (ppm) map (computed)**
	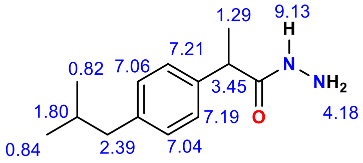	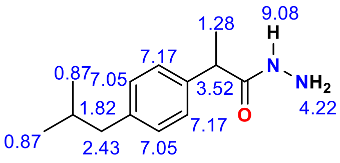
**HL**	**δ (ppm)**	
	11.12(1H, s, OH), 10.03(1H, br, NH), 8.39(1H, s, CH), 7.27-6.63(8H, m, ArH), 3.69(2H, s, CH_2), 2.41(2H, s, CH_2), 1.83(H, s, CH), 1.39(3H, m, CH_3), 0.87-0.82(6H, m, $(CH_3)_2$).	
	**Δ (ppm) map (experimental)**	**δ (ppm) map (computed)**
	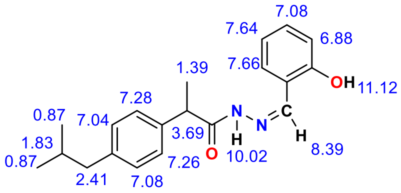	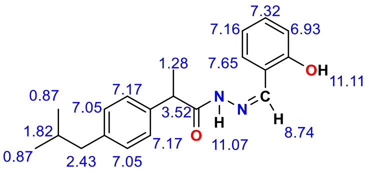

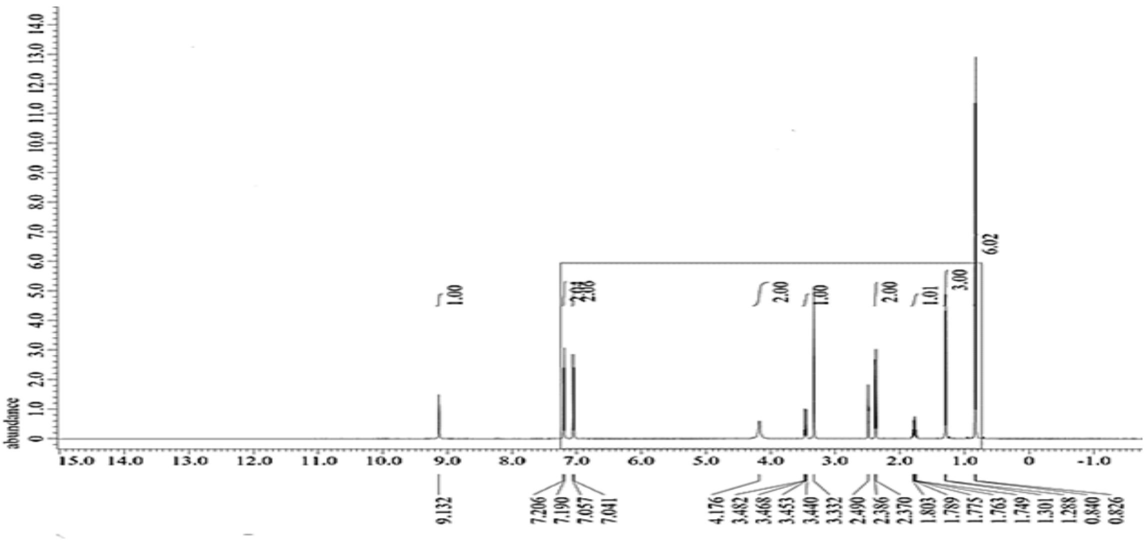

Figure S1: ^{1}HNMR spectrum of Hydrazide Ibuprofen **(HI)**.

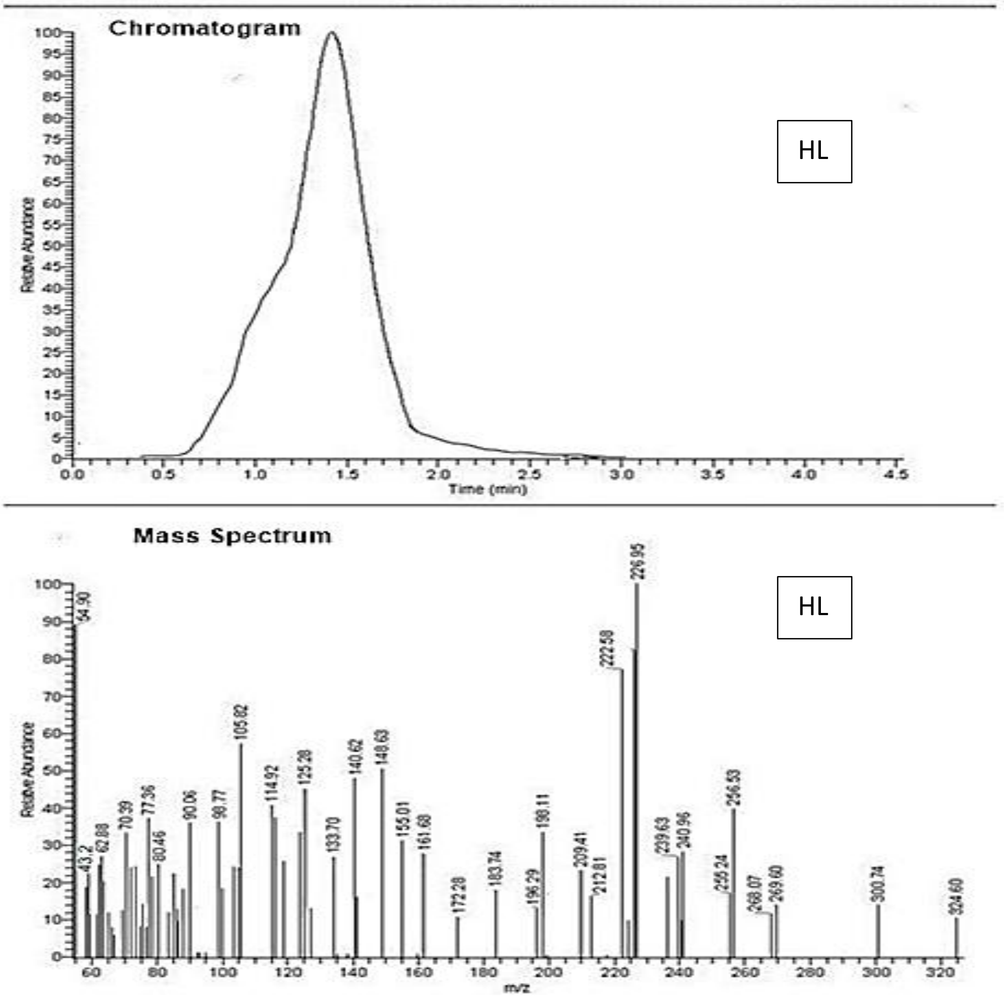

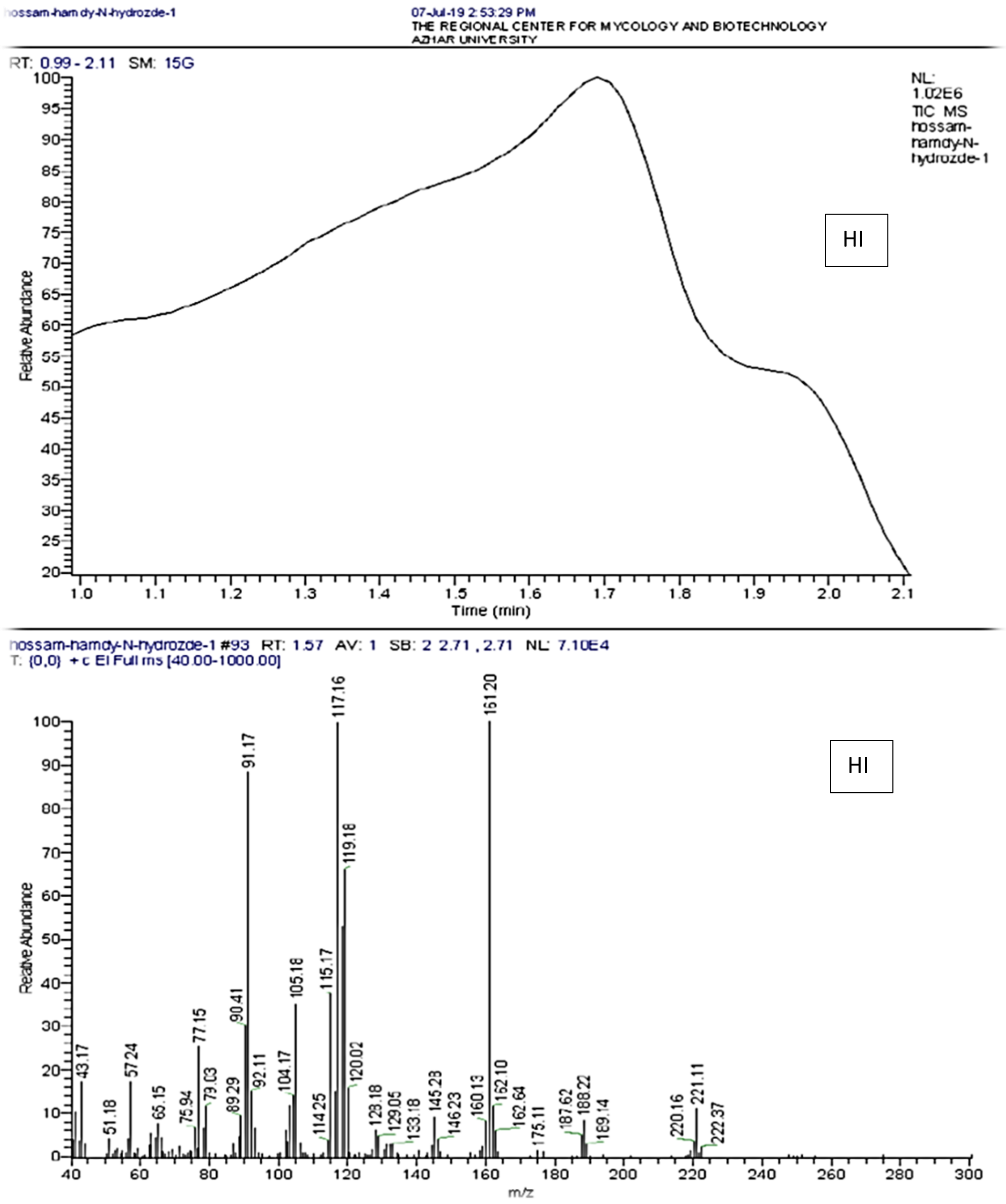

Figure S2: Chromatogram and mass spectrum of the Ibuprofen hydrazone **(HL)** and hydrazide **(HI).**

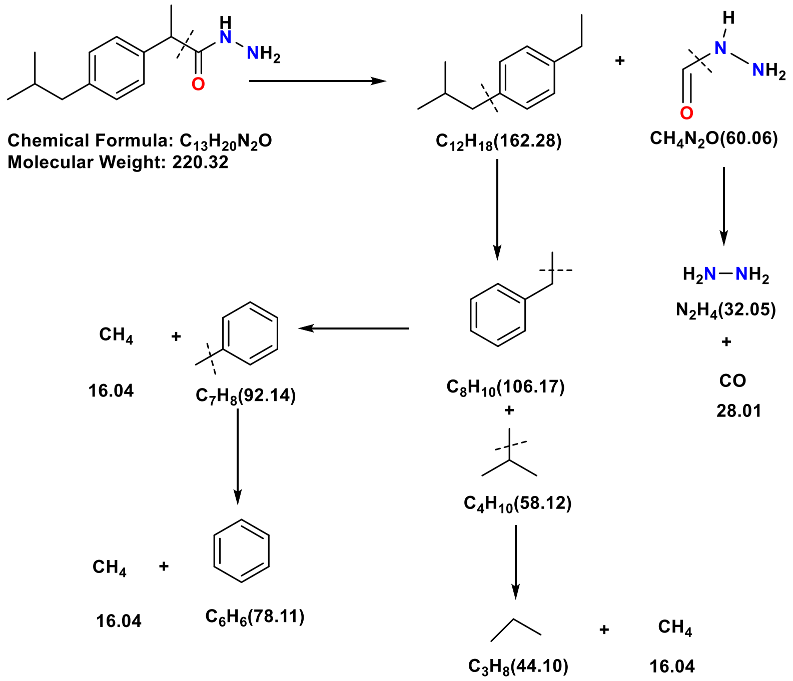

Scheme S1: The chromatogram of pathway fragmentation of Hydrazide Ibuprofen (**HI**).

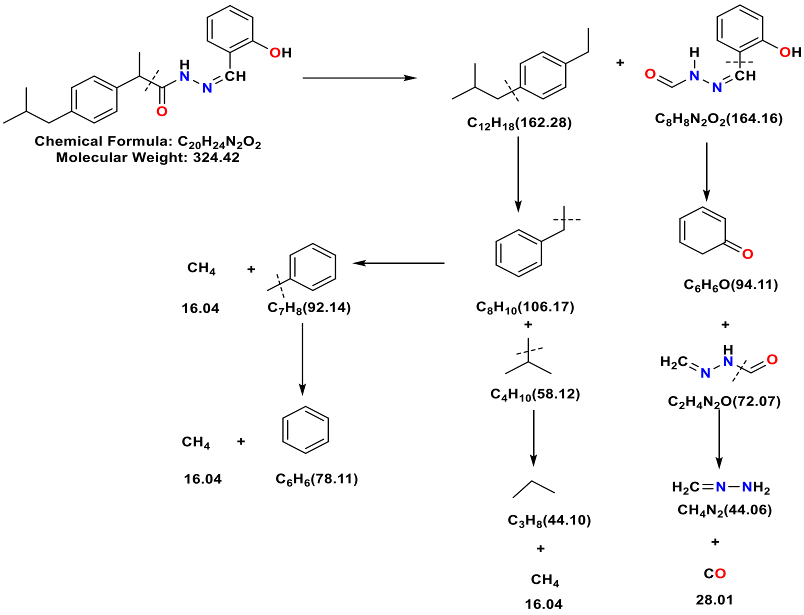

Scheme S2: The chromatogram of pathway fragmentation of **HL** ligand**.**

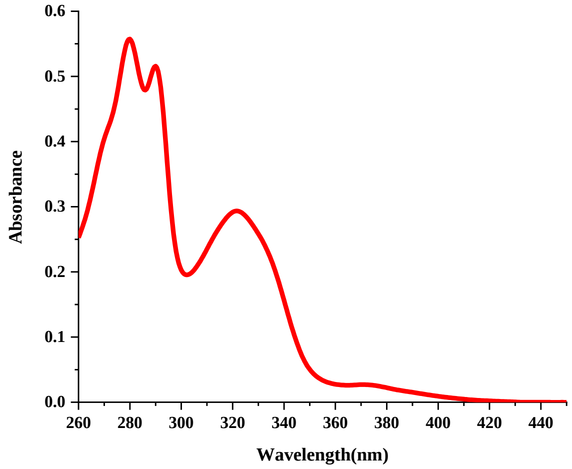

Figure S3: UV-Vis. Spectrum of the ligand **HL**.

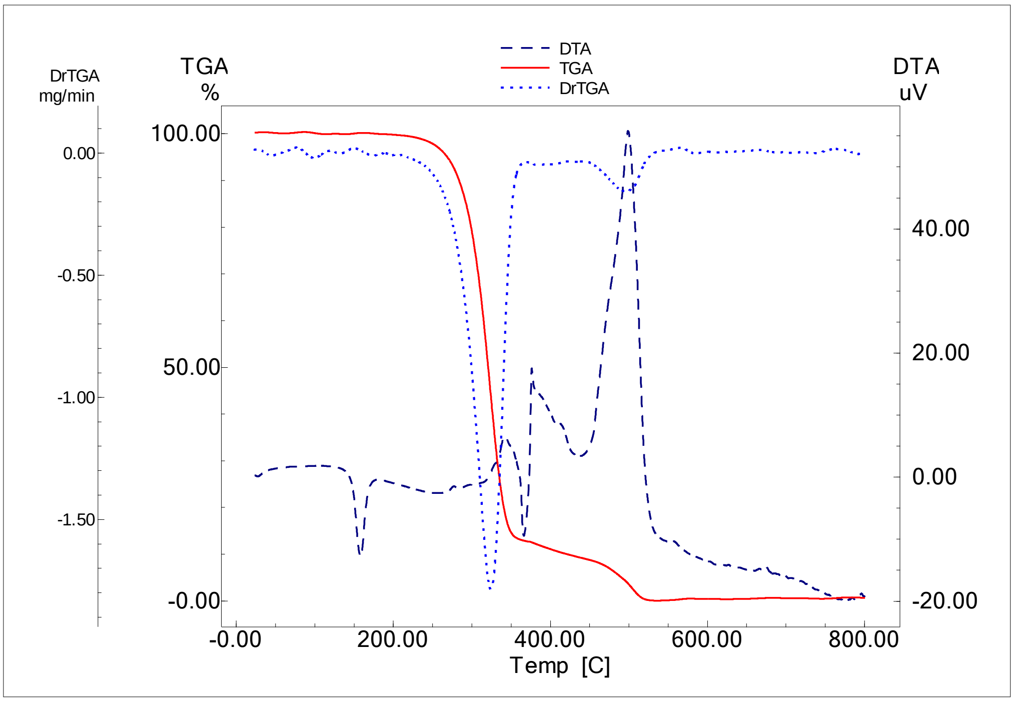

Figure S4: Thermal decomposition of Schiff base **HL.**

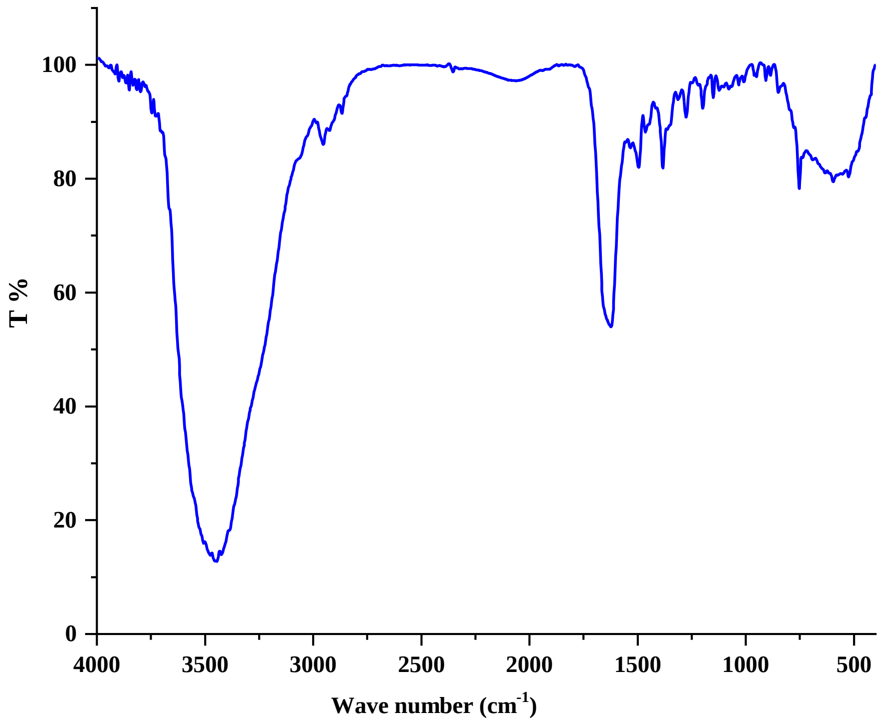

Figure S5: FTIR spectrum of $[Cu(L)(H_2O)]Cl.2H_2O$ complex.

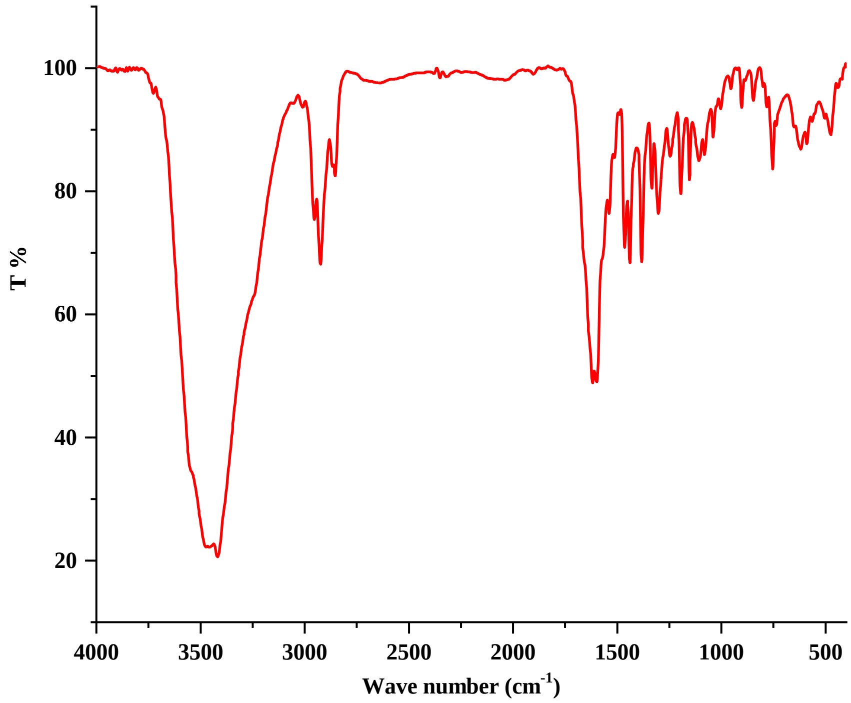

Figure S6: FTIR spectrum of $[Ni(L)_2]$ complex.

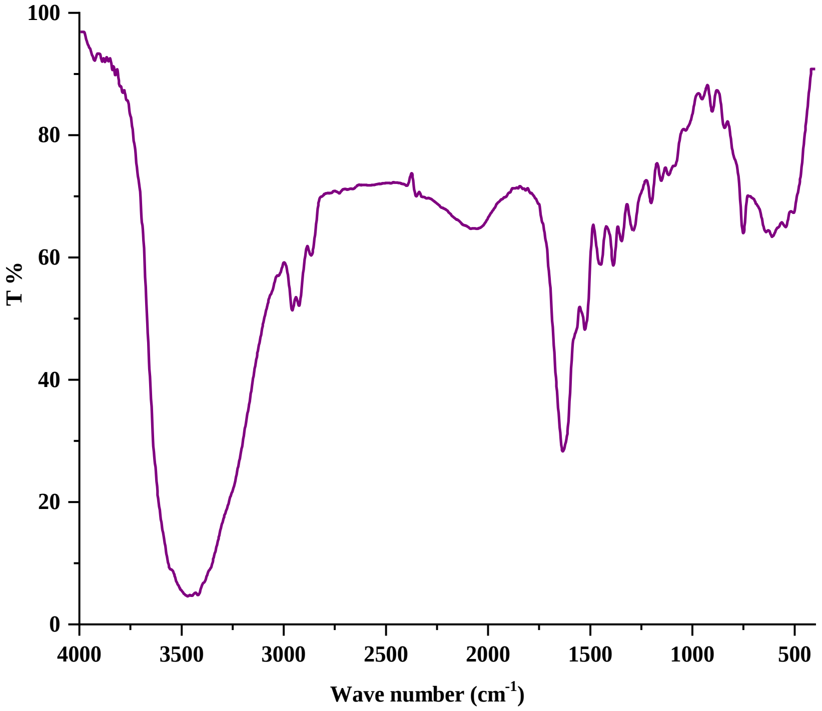

Figure S7: FTIR spectrum of [Co(L)$_2$]H_2O complex.

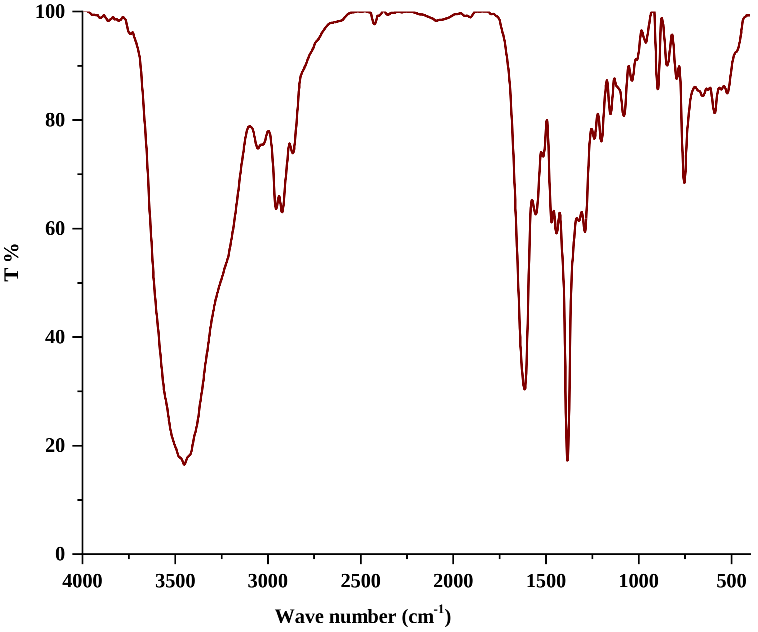

Figure S8: FTIR spectrum of [Gd(L)$_2$(H_2O)$_2$] (NO_3).3H_2O complex.

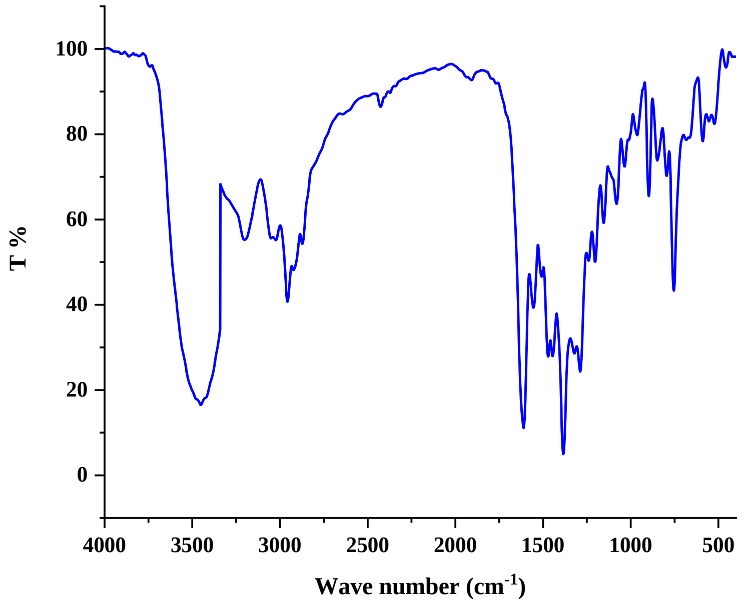

Figure S9: FTIR spectrum of $[Sm(L)_2(H_2O)_2]$ $(NO_3).2H_2O$ complex.

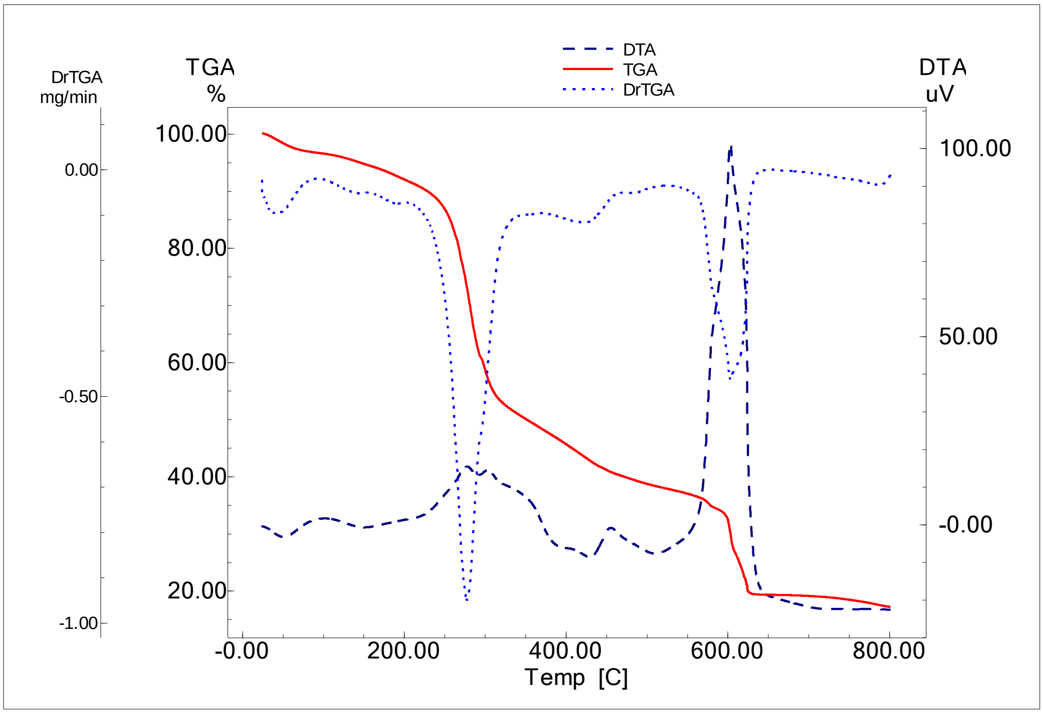

Figure S10: TGA/TG and DTA curves of $[Cu(L)(H_2O)]Cl.H_2O$ complex.

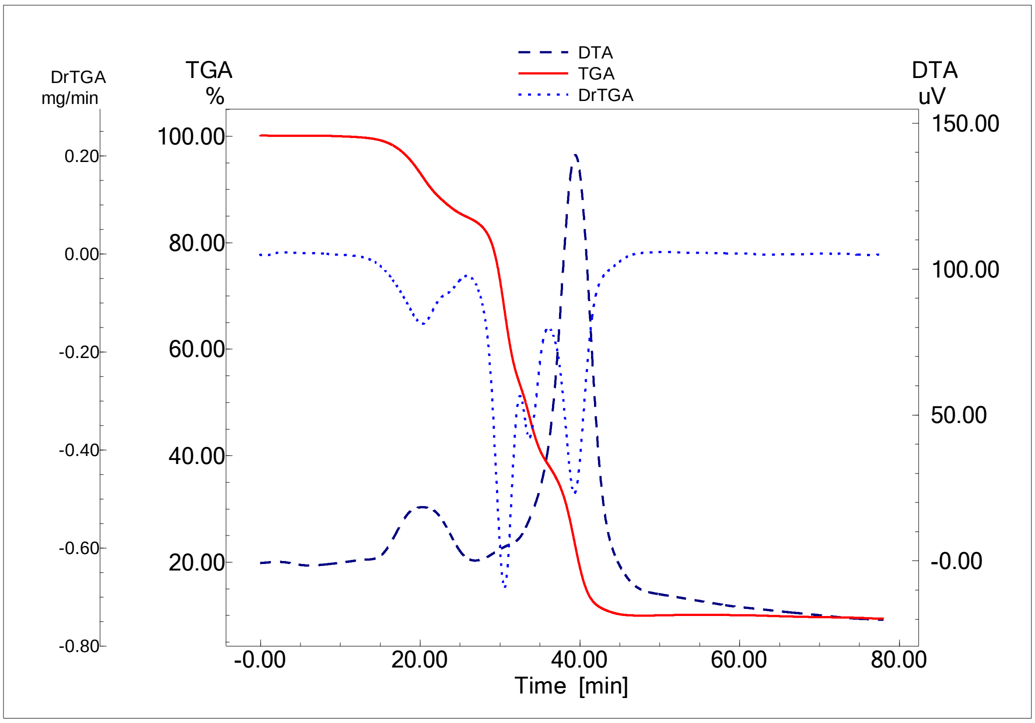

Figure S11: TGA/TG and DTA curves of $[Ni(L)_2]$ complex.

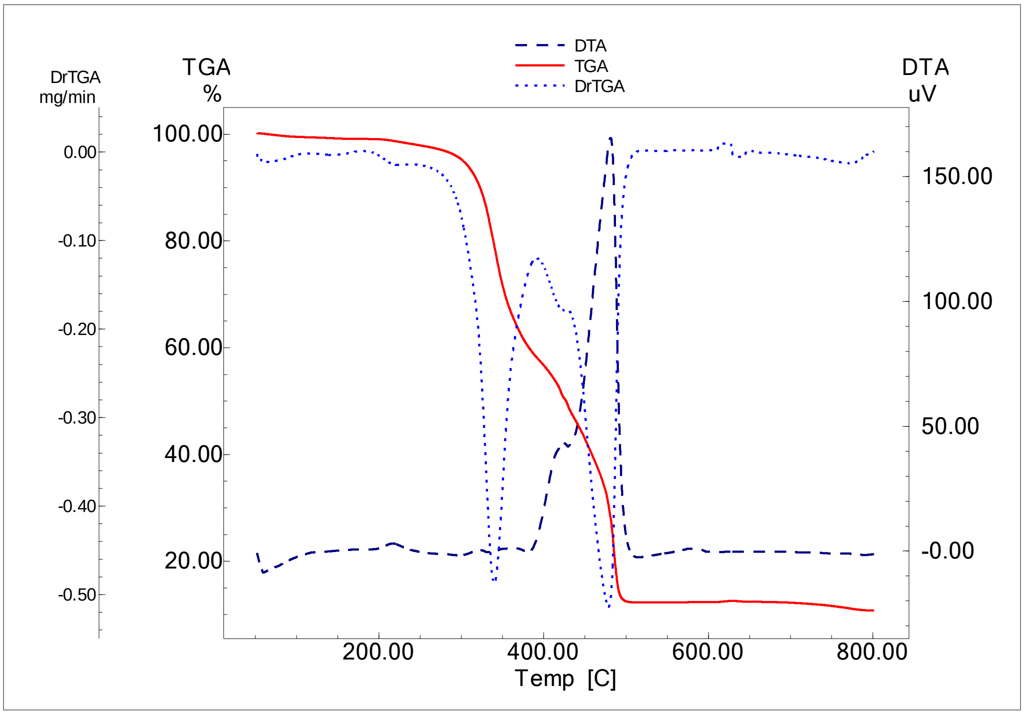

Figure S12: TGA/TG and DTA curves of $[Co(L)_2]1/2H_2O$ complex.

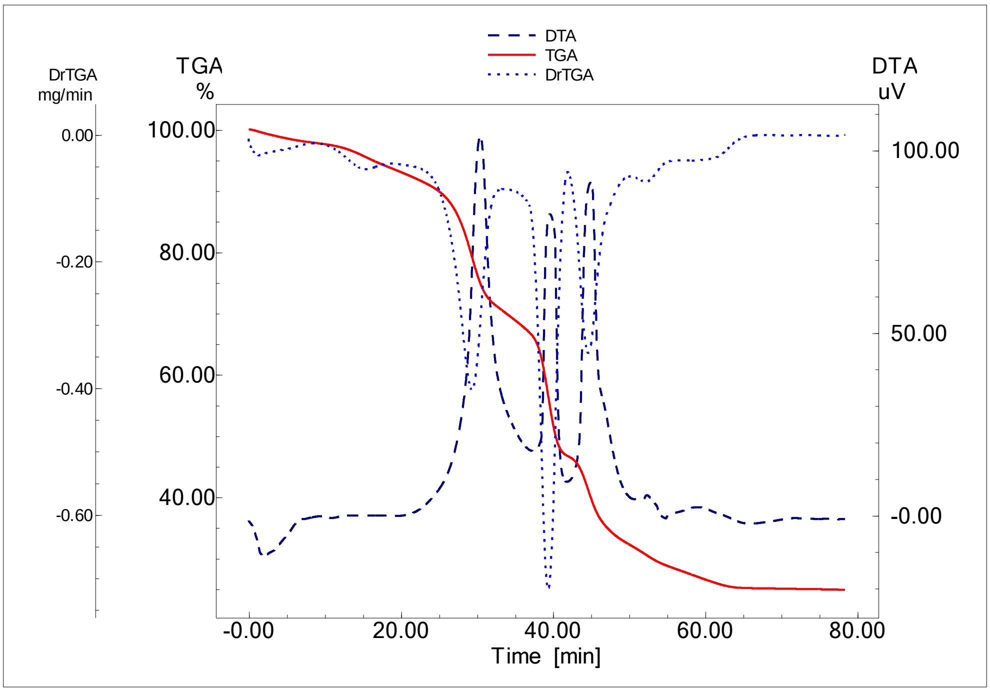

Figure S13: TGA/TG and DTA curves of $[Sm(L1)_2(H_2O)_2](NO_3).2H_2O$ complex.

Table S2: DTA of Complexes derived from HL Schiff base.

Compound	*Temp. range ⁰C*	*DTA peak temp ⁰C*	*Peak type*	*ΔH (KJ/g)*	*Process*
$[Cu(L)(H_2O)]Cl\cdot H_2O$	28-156	55	Endo	0.058	Dehydration+ Coordination sphere
	160-514	278	Exo	-0.997	ligand degradation + Coordination sphere
	520-800	603	Exo	-2.24	Final degradation
$[Ni(L)_2]$	119-300	235	Exo	-1.21	Ligand degradation
	311-786	449	Exo	-6.17	End degradation
$[Co(L)_2]\cdot 1/2H_2O$	55-150	60	Endo	0.311	Dehydration
	156-278	218	Exo	-0.109	ligand degradation
	435-495	481	Exo	-7.9	Final degradation
	24-210	116	Endo	0.231	Dehydration

$[Gd(L)_2(H_2O)_2(NO_3)](NO_3)_2\cdot 3/2H_2O$	307-436	344, 412	Exo	-3.48	Coordination sphere+ Partial Ligand degradation
	436-550	509	Exo	-0.698	Ligand degradation
	569-727	627	Exo	-1.01	Final degradation
$[Sm(L)_2(H_2O)_2(NO_3)](NO_3)_2\cdot 2H_2O$	65-182	128	Endo	0.099	Dehydration + Coordination sphere
	320-350	345	Exo	-2.21	Coordination sphere+ Ligand degradation
	440-469	446	Exo	-0.545	Ligand degradation
	474-486	492,554	Exo	-1.14	Final degradation

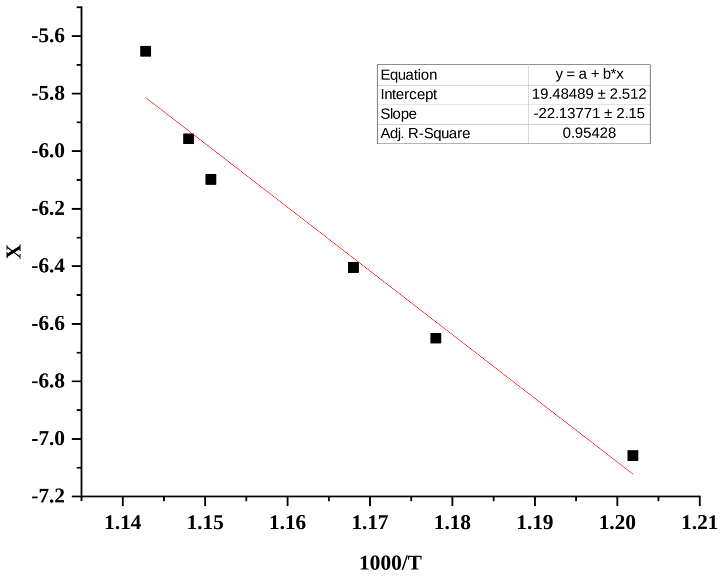

Figure **S14**: The fit-linear curve of the liberation of coordinate water step of **Cu-L1** complex.

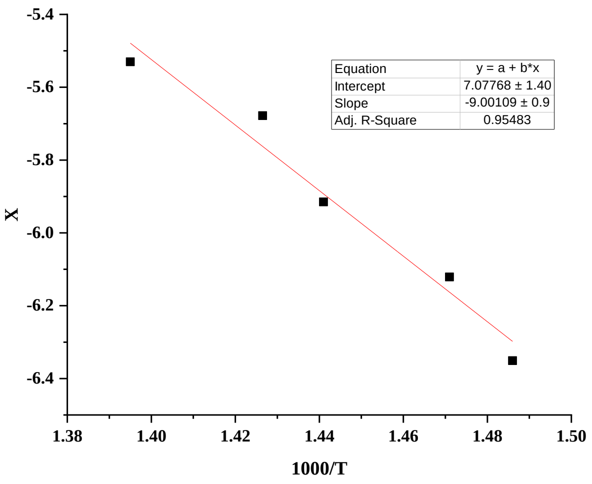

Figure S15: Fit-linear curve of liberation of coordinate water step of **Ni-L1** complex.

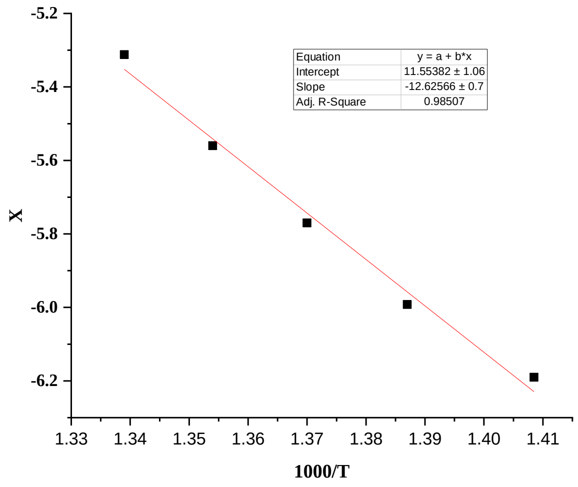

Figure S16: Fit-linear curve of liberation of coordinate water step of **Co-L1** complex.

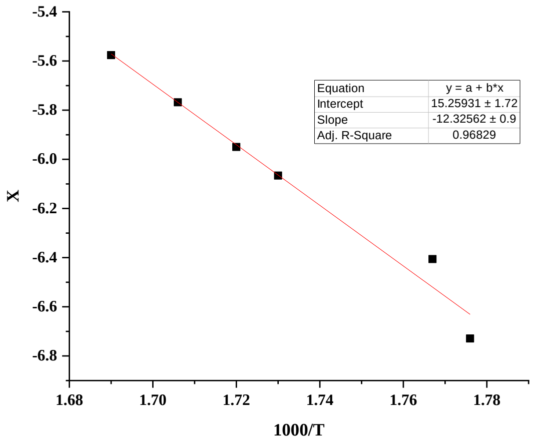

Figure S17: Fit-linear curve of liberation of coordinate water step of **Gd-L1** complex.

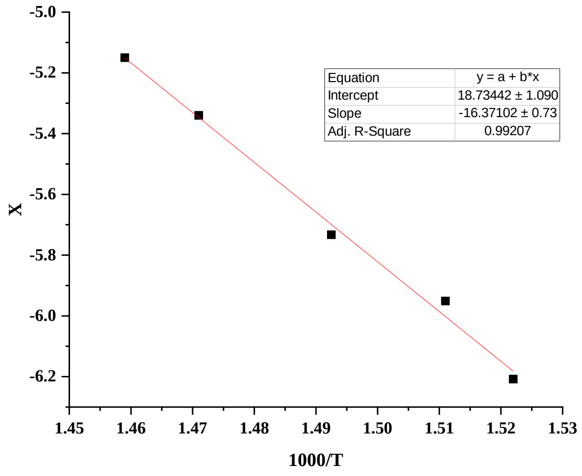

Figure S18: Fit-linear curve of liberation of coordinate water step of **Sm-L1** complex.

Table S3: The electronic spectra and magnetic features of the **HL** ligand and its complexes.

Compound	***Peak***		***ε**** ***($M^{-1}cm^{-1}$)*** x10^4	***Assignment***	***10Dq***		***μeff (μ_B)***	***Postulated Structure***
	nm	***cm^{-1}***			***cm^{-1}***	***kJ/mol***		
HL	280	35714	5.57	$\pi \rightarrow \pi^*$	---	---	---	---
	290	34482	5.12					
	322	31055	2.94	$n \rightarrow \pi^*$				
L-Cu	292	34246	2.27	$\pi \rightarrow \pi^*$	16556	201	1.49	Square planar
	363	27548	2.7	$n \rightarrow \pi^*$				
	404	24752	2.78	CT				
	451	22172	11.86					
	600	16556	0.05	d-d				
L-Ni	317	31545	13.72	$n \rightarrow \pi^*$	24875	302	3.24	Distorted octahedral
	400	25188	11.88	CT				
	628	24875	9.25	$^3A_{2g} \rightarrow {}^1E_g$				
L-Co	290	34482	4.43	$\pi \rightarrow \pi^*$	19762	240	5.00	Octahedral
	400	25000	4.58	$n \rightarrow \pi^*$				
	407	24570	4.56	$n \rightarrow \pi^*$				
	450	22222	3.13	CT				
	506	19762	15.87	$^4T1g(F) \rightarrow {}^4T2g(P)$				
L-Gd	291	34364	3.21	$\pi \rightarrow \pi^*$	---	---	8.97	Bicapped trigonal prismatic / Square antiprismatic
	402	24875	3.25	$n \rightarrow \pi^*$				
	452	22123	4.018	CT				
	504	19841	2.99					
L-Sm	239	41841	16.32	$\pi \rightarrow \pi^*$	---	---	1.33	Bicapped trigonal prismatic / Square antiprismatic
	347	28818	3.41	$n \rightarrow \pi^*$				

* ***ε*= Absorptivity, 10^{-5} M in DMSO, $M^{-1}cm^{-1}$.

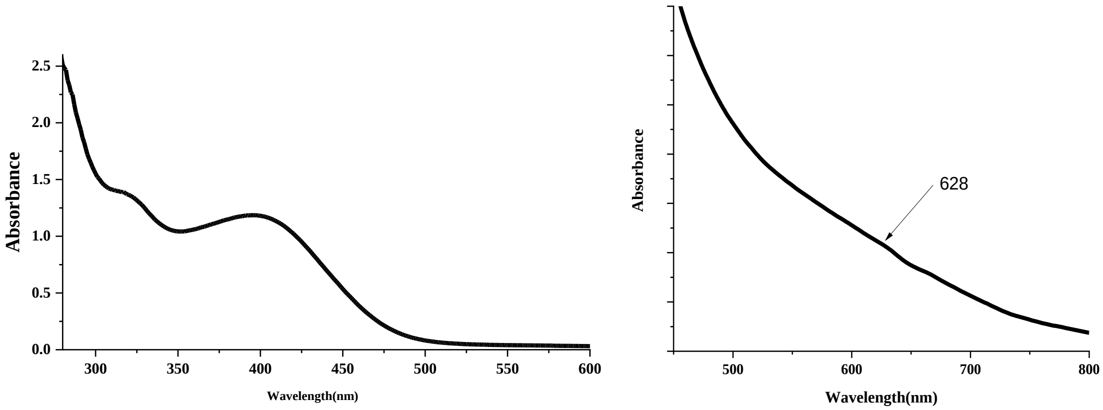

Figure S19: UV-Vis. spectrum of L1-Ni.

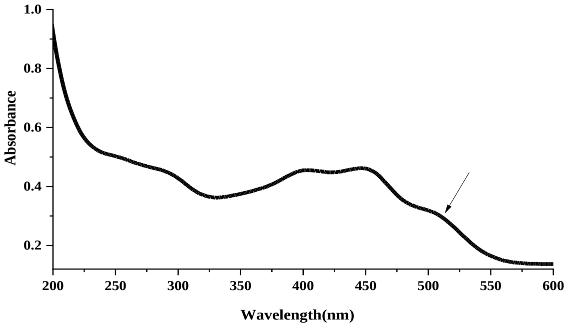

Figure S20: UV-Vis. spectrum of L1-Co.

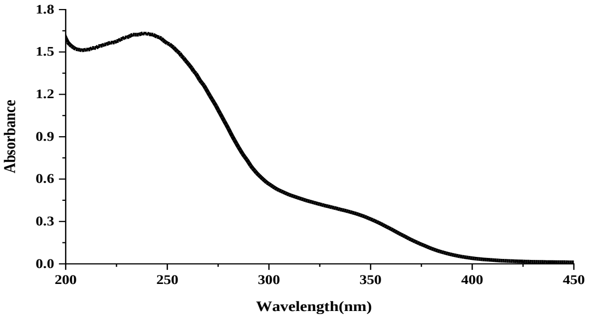

Figure S21: UV-Vis. spectrum of L1-Sm.

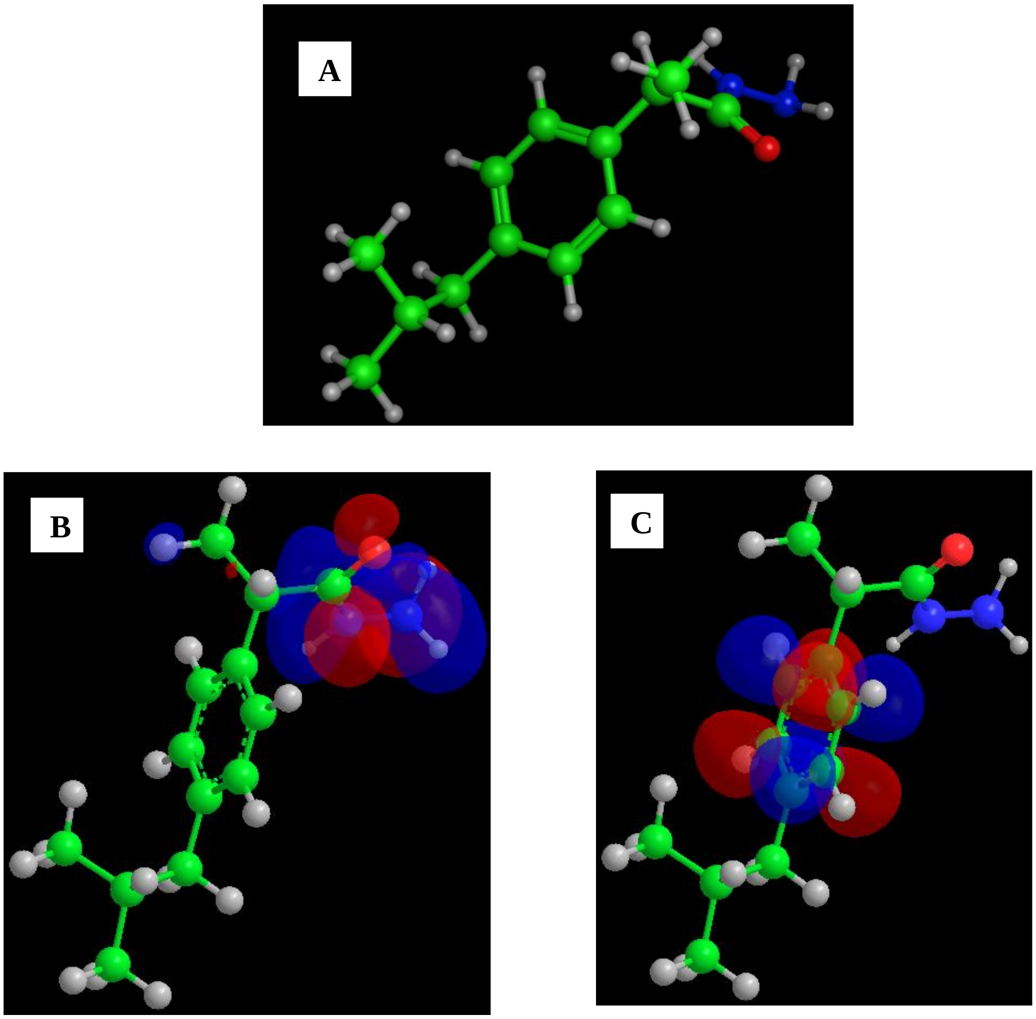

Figure S22: The DFT simulation for the Hydrazide Ibuprofen. **(A)** 3D view, **(B)** HOMO and **(C)** LUMO.

Table S4: The data from DFT calculations and the properties of synthesized compounds.

COMPOUND	IBUPROFEN	HI	HL
FORMULA	$C_{13}H_{18}O_2$	$C_{13}H_{20}N_2O$	$C_{20}H_{24}N_2O_2$
ATOMS	33	36	**48**
ORBITALS	78	84	**120**
ELECTRONS	82	88	**126**
SCF ENERGY	-93.85 au	-98.24 au	**-144.94 AU**
DIPOLE	1.94 d	3.37 d	**6.20 D**
E_{LUMO}	0.007	0.0036	**-0.012**
E_{HOMO}	-0.349	-0.351	**-0.325**
$E_{LUMO-HOMO}$	127.81 nm	128.56 nm	**145.36 NM**
I (I.E)	0.348 au	0.351 au	**0.325 AU**
ELECTRON AFFINITY (A)	-0.007 au	-0.004 au	**0.012 AU**
ABSOLUTE ELECTRONEGATIVITY(X)	0.138	0.174	**0.168**
ABSOLUTE HARDNESS ( )	0.211	0.177	**0.157**
ABSOLUTE SOFTNESS ( )	4.739	5.640	**6.382**
GLOBAL SOFTNESS (S)	2.370	2.820	**3.191**
GLOBAL ELECTROPHILICITY ( )	0.045	0.085	**0.090**
CHEMICAL POTENTIAL (PI)	-0.138	-0.174	**-0.168**
ADDITIONAL ELECTRONEGATIVITY (NMAX)	0.654	0.980	**1.073**
TOXIC.	No	Yes	**NO**
RSYNTH (%)	66.67	75	**79.17**
WEIGHT (G/MOL)	206.28	220.32	**324.42**
TPSA	37.30<br>Hd 1 Ha 2	55.12<br>Hd 2 Ha 2	**61.69**<br>Hd 2 **HA 3**
LOG P	3.07	1.98	**3.84**
LOG S	**-3.64**	**-3.90**	**-5.46**

* **SCF energy**: self-consistent field, **I**: Ionization potential, **Rsynth (%)**: Resynthesized %, **TPSA**: topological polar surface area, **HD**: Hydrogen donor, **HA**: Hydrogen acceptor, **Log p**: lipophilicity parameter and **Log S**: water solubility parameter.

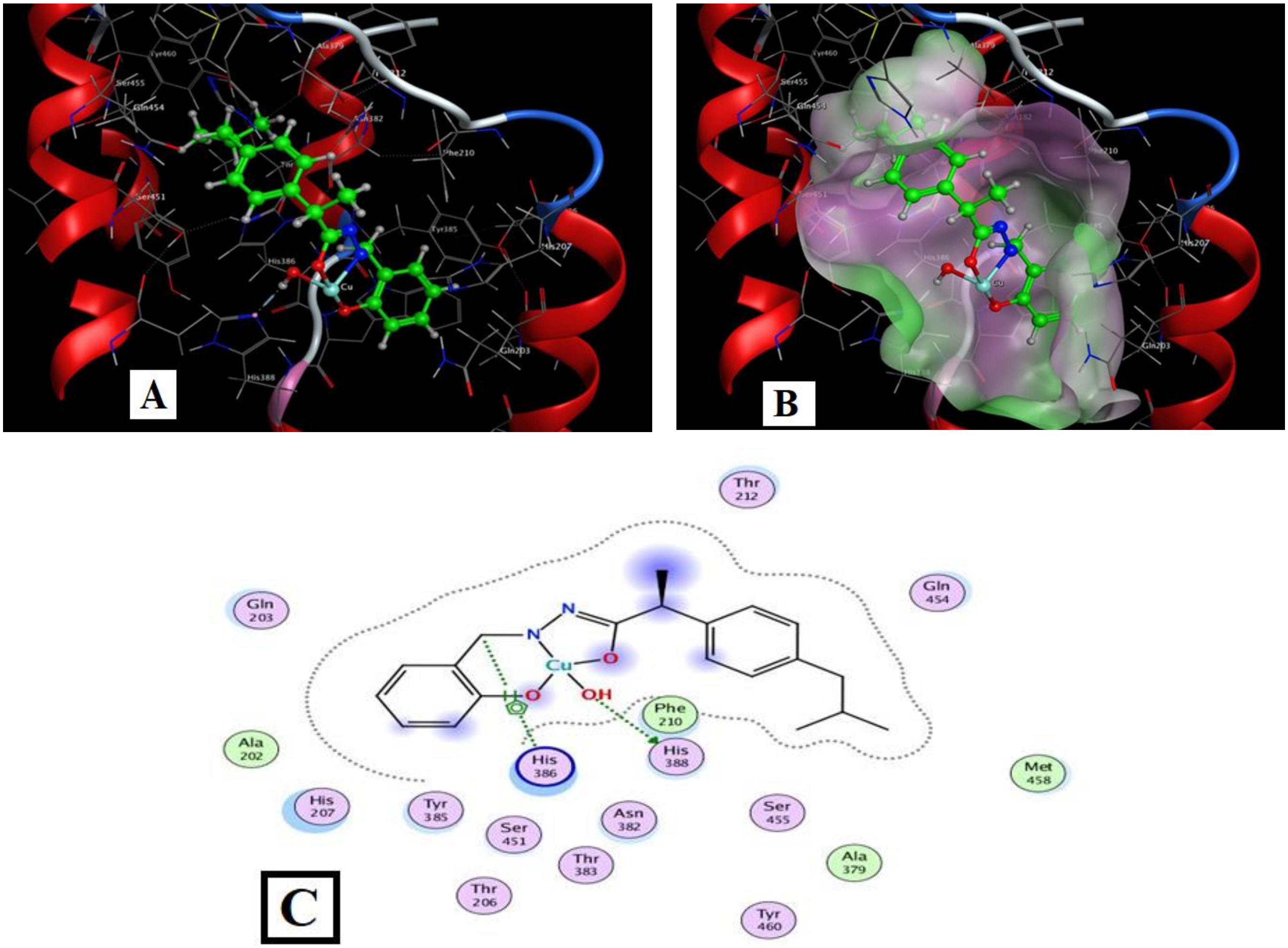

Figure S23: Docking model of the interaction of **Cu-L1** with ***Cox2*** [PDB code: 5IKT] bonding sites:

(A) 3D interaction diagram (B) The surface properties [Hydrophilic sites (violet color), neutral sites (white color) and lipophilic sites (green color)]. (C) 2D interaction diagram

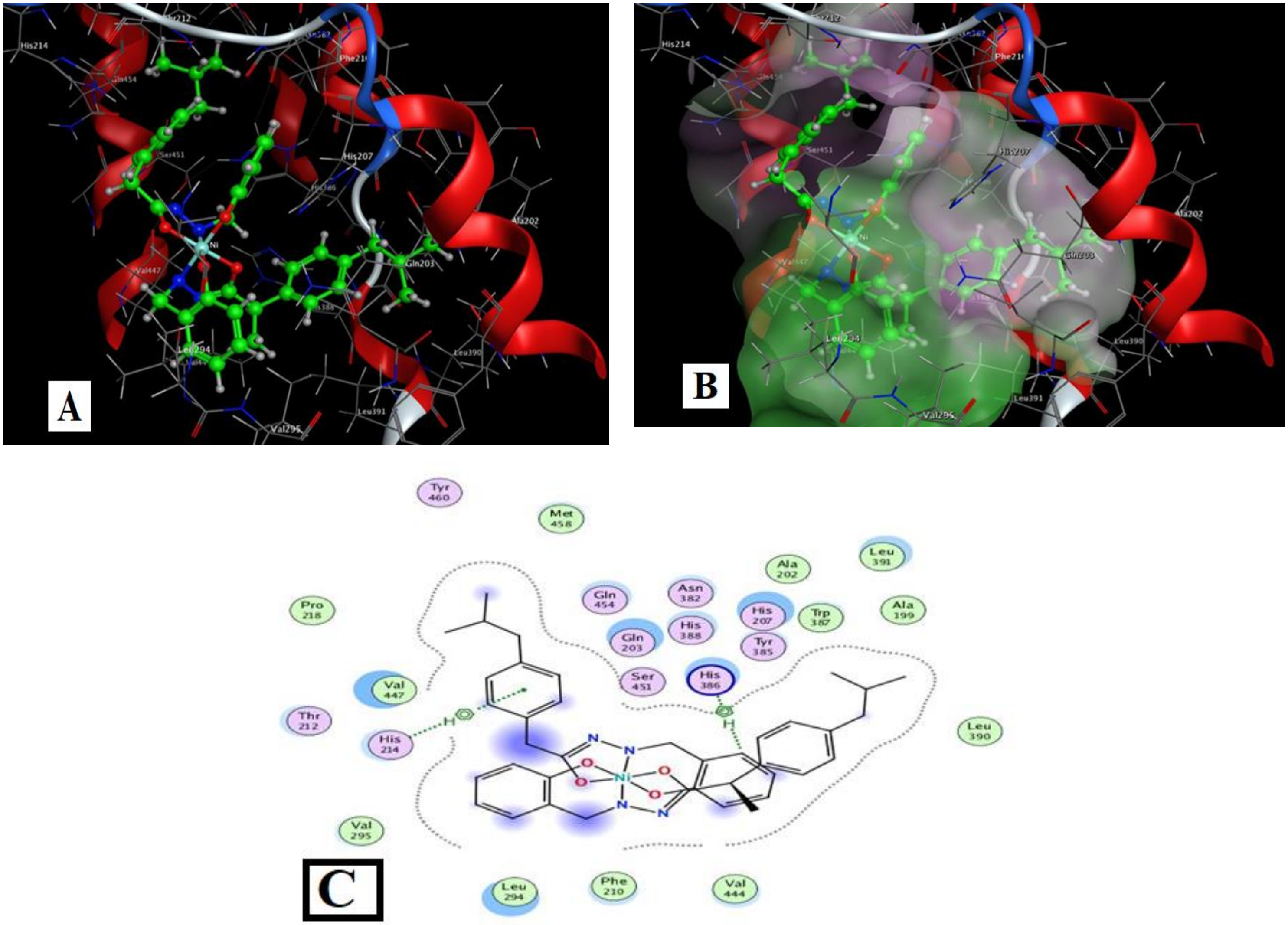

Figure S24: Docking model of the interaction of **Ni-L1** with ***Cox2*** [PDB code: 5IKT] bonding sites:

(A) 3D interaction diagram (B) The surface properties [Hydrophilic sites (violet color), neutral sites (white color) and lipophilic sites (green color)]. (C) 2D interaction diagram

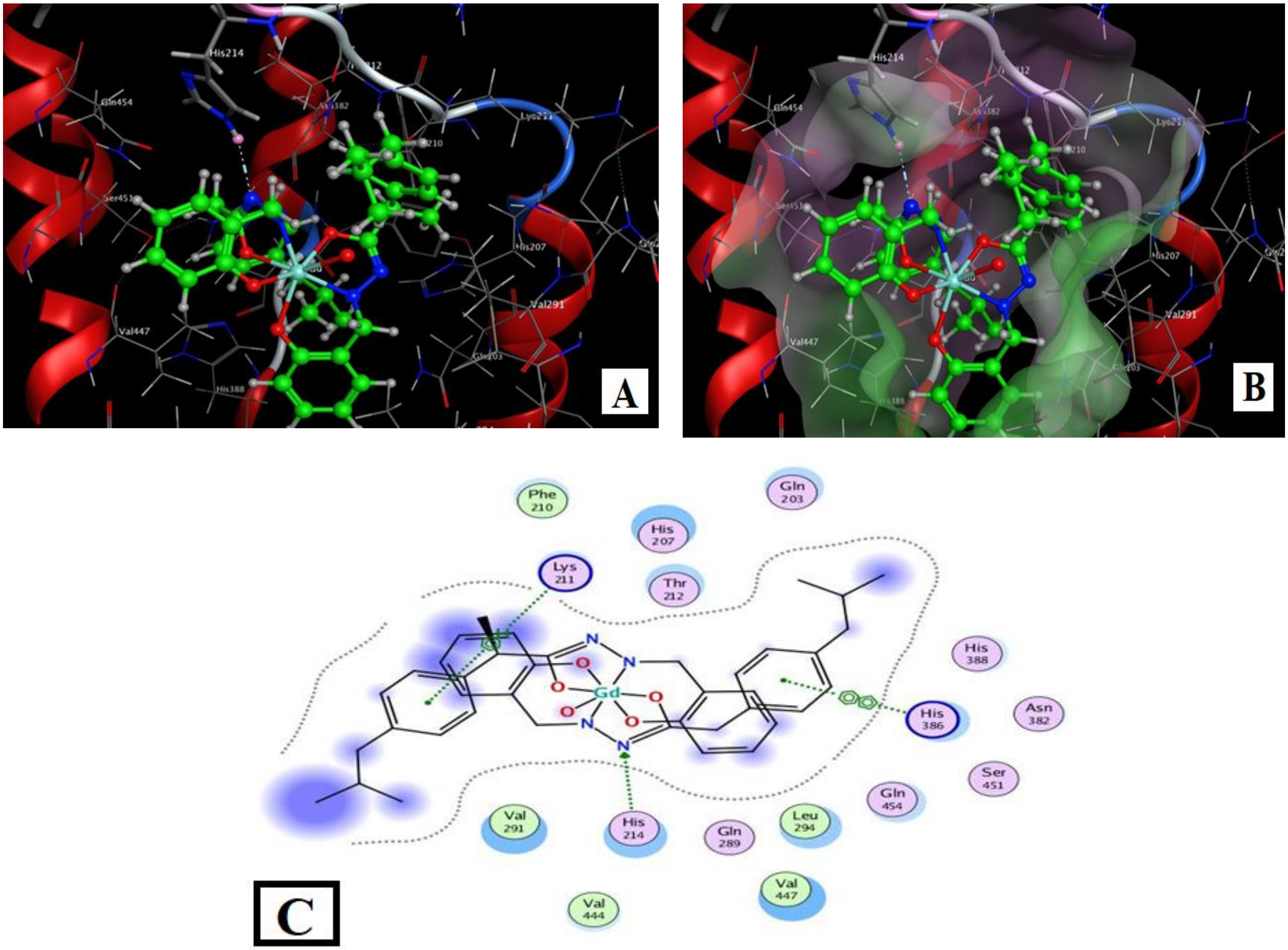

Figure S25: Docking model of the interaction of **Gd-L1** with ***Cox2*** [PDB code: 5IKT] bonding sites:

(A) 3D interaction diagram (B) The surface properties [Hydrophilic sites (violet color), neutral sites (white color) and lipophilic sites (green color)]. (C) 2D interaction diagram

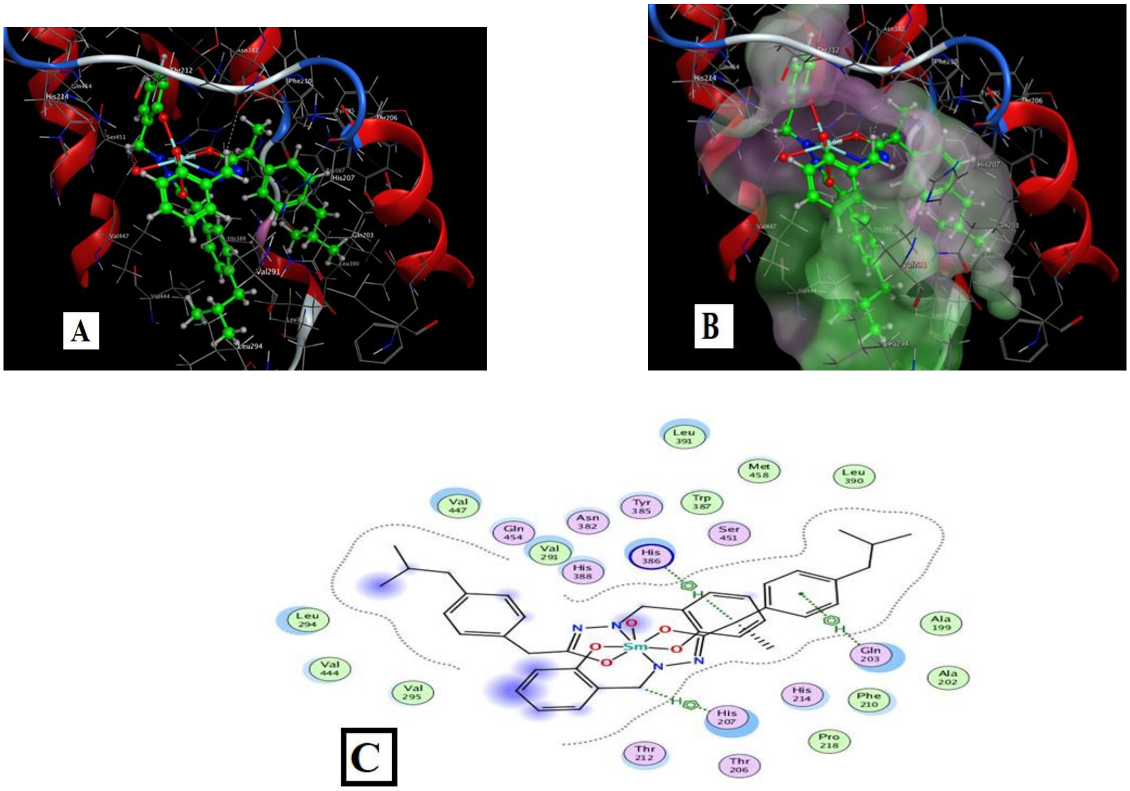

Figure S26: Docking model of the interaction of **Sm-L1** with ***Cox2*** [PDB code: 5IKT] bonding sites:

(A) 3D interaction diagram (B) The surface properties [Hydrophilic sites (violet color), neutral sites (white color) and lipophilic sites (green color)]. (C) 2D interaction diagram

Table S5: In vitro COX-1 and COX-2 inhibition of the synthesized derivatives.

**Compound**	**COX-2** ***IC*₅₀ *(μM)*** [a]
**Hydrazide**	4.3
**Schiff base (L)**	4.9
**Cu-L**	5.6
**Ni-L**	3.7
**Co-L**	1.7
**Gd-L**	2.3
**Sm-L**	2.9
**Ibuprofen**	31.4
**Indomethacin**	0.1
**Diclofenac sodium**	0.8
